# Supplementary material for: Simultaneous Screening of 322 Residual Pesticides in Fruits and Vegetables Using GC-MS/MS and Deterministic Health Risk Assessments
Source: Foods. 2023 Aug 9;12(16):3001. doi: 10.3390/foods12163001 (PMC10453053; doi:10.3390/foods12163001)
Supplement: Supplementary file 1 [file foods-12-03001-s001.zip › foods-2497950-supplementary.pdf]

Supporting information

Simultaneous Screening of 322 Residual Pesticides in Fruits and Vegetables Using GC-MS/MS and Deterministic Health Risk Assessments

Byong-Sun Choi [1,†], Dong-Uk Lee [2,†], Woo-Seong Kim [3], Chan-Woong Park [3], Won-Jo Choe [4,*] and Myung-Jun Moon [1,*]

1 Department of Industrial Chemistry, Pukyong National University, Busan 48513, Republic of Korea; sunny2597@naver.com
2 Biomedical Manufacturing Technology Center, Korea Institute of Industrial Technology, Yeongcheon 38822, Republic of Korea; young2home@pukyong.ac.kr
3 Center of Food & Drug Analysis, Busan Regional Office of Food and Drug Safety, Ministry of Food and Drug Safety, Busan 47537, Republic of Korea; kwsh1964@korea.kr (W.-S.K.); pcw0324@korea.kr (C.-W.P.)
4 Pesticides & Veterinary Drug Residues Division, National Institute of Food & Drug Safety Evaluation,
Ministry of Food and Drug Safety, Cheongju 28159, Republic of Korea
* Correspondence: aragaya06@korea.kr (W.-J.C.); mjmoon@pknu.ac.kr (M.-J.M.)
† These authors contributed equally to this work.

Table S1. Pesticides that are mainly used in Korean agriculture by establishing residual acceptance standards in Korea.

No.	Pesticide Components	Setting Criteria	Not setting criteria
1	2,6-Diisoporpylnaphthalene		X
2	Acetochlor	O	
3	Acibenzola_s_methyl	O	
4	Acrinathrin	O	
5	Alachlor	O	
6	Aldrin	O	
7	Allethrin		X
8	Allidochlor		X
9	Ametryn		X
10	Anilofos		X
11	Aramit		X
12	Aspon		X
13	Atrazine		X
14	Azaconazole		X
15	Azinphos-ethyl		X
16	Azinphos-methyl		X
17	Benalaxyl	O	
18	Benodanil		X
19	Benoxacor		X
20	Benzoylprop_ethyl		X
21	BHC(α-, β-, δ-)	O	
22	Lindane(γ-BHC)	O	
23	Bifenox	O	
24	Bifenthrin	O	
25	Bromacil	O	
26	Bromobutide	O	
27	Bromophos-ethyl		X
28	Bromophos-methyl		X
29	Cinidoneethyl		X
30	Cinmethylin		X
31	Clomazon	O	
32	Clomeporp		X
33	Coumaphos		X
34	Crotoxyphos		X
35	Cyanazine		X

36	Cyanophos		X
37	Cycloate		X
38	Cyflufenamid	O	
39	Cyfluthrin	O	
40	Cyhalofop-butyl	O	
41	Cyhalothrin	O	
42	Cypermethrin	O	
43	Cyprazine		X
44	Cyproconazole	O	
45	Cyprodinil	O	
46	DDT	O	
47	Deltamethrin	O	
48	Demeton_O		X
49	Demeton_S	O	
50	Demeton_S_methylsulfone		X
51	Desmetryn		X
52	Endrin	O	
53	EPN		X
54	Epoxiconazole	O	
55	EPTC		X
56	Esprocarb	O	
57	Etaconazole		X
58	Ethalfluralin	O	
59	Ethion	O	
60	Ethofumesate		X
61	Ethoprophos	O	
62	Ethychlozate	O	
63	Etofenprox	O	
64	Etoxazole	O	
65	Etridiazole	O	
66	Etrimfos	O	
67	Fenamidone	O	
68	Fenamiphos		X
69	Fenarimol	O	
70	Fenazaquin	O	
71	Fenbuconazole	O	
72	Fenchlorphos		X

73	Fenclorim	O	
74	Fenfuram		X
75	Fenitrothion	O	
76	Fenobucarb	O	
77	Fenothiocarb		X
78	Fenoxanil	O	
79	Fenoxycarb		X
80	Fenpropathrin	O	
81	Fenson		X
82	Fenthion	O	
83	Fenvalerate	O	
84	Fipronil	O	
85	Imazalil	O	
86	Indanofan	O	
87	Indoxacarb	O	
88	Iprobenfos	O	
89	Iprodione	O	
90	Iprovalicarb	O	
91	Isazofos		X
92	Isofenphos	O	
93	Isofenphos-methyl		X
94	Isopropalin		X
95	Isoprothiolane	O	
96	Isotianil	O	
97	Isoxadifen-ethyl		X
98	Isoxanthion		X
99	Kresoxim-methyl	O	
100	Lactofen		X
101	Leptophos		X
102	Malathion	O	
103	Mecarbam	O	
104	Mefenacet	O	
105	Mefenpyr-diethyl		X
106	Mepronil	O	
107	Metazachlor	O	
108	Metconazole	O	
109	Methidathion	O	

110	Methoprotryne		X
111	Methoxychlor		X
112	Methyltrithion		X
113	Metolachlor	O	
114	Metrafenone	O	
115	Metribuzin	O	
116	MGK-264		X
117	Mirex		X
118	Piperonyl butoxide	O	
119	Piperophos		X
120	Pirimiphos-ethyl		X
121	Pirimiphos-methyl	O	
122	Pretilachlor	O	
123	Primicarb	O	
124	Probenazole	O	
125	Prochloraz	O	
126	Procymidone	O	
127	Profenofos	O	
128	Profluralin		X
129	Prometon		X
130	Prometryn		X
131	Pronamide	O	
132	Propachlor		X
133	Propanil	O	
134	Propazine		X
135	Propetamphos		X
136	Propham		X
137	Propiconazole	O	
138	Propisochlor		X
139	Prothiophos		X
140	Pyracabolid		X
141	Pyraclofos		X
142	Pyrazophos		X
143	Pyridaben	O	
144	Pyridalyl	O	
145	Pyridaphenthion		X
146	Pyrifenox		X

147	Pyrimidifen		X
148	Pyriminobac-methyl	O	
149	Quinalphos		X
150	Quinoxyfen	O	
151	Triadimefon	O	
152	Triadimenol	O	
153	Triallate		X
154	Triazophos	O	
155	Tribufos		X
156	Tridiphane		X
157	Triflumizole	O	
158	Bromopropylate		X
159	Bupirimate		X
160	Butachlor	O	
161	Butafenacil		X
162	Butralin		X
163	Butylate		X
164	Cadusafos	O	
165	Captan	O	
166	Carbophenothion		X
167	Chinomethionat	O	
168	Chlorbenside		X
169	Chlorbufam		X
170	Chlordane	O	
171	Chlorethoxyfos		X
172	Chlorfenapyr	O	
173	Chlorfenson		X
174	Chlorfluazuron	O	
175	Chlorflurenol_methyl		X
176	Chlornitrofen		X
177	Chlorobenzilate		X
178	Chloroneb		X
179	Chloropropylate		X
180	Chloroxuron		X
181	Chlorpropham	O	
182	Chlorpyrifos	O	
183	Chlorpyrifos-methyl	O	

184	Chlorthal-dimethyl		X
185	Chlorthalonil	O	
186	Chlorthion		X
187	Chlorthiophos		X
188	Chlozolinate		X
189	Diafor		X
190	Diallate		X
191	Diazinon	O	
192	Dichlofenthion		X
193	Dichlofluanid		X
194	Dichlormid		X
195	Dichlorvos	O	
196	Diclobutrazole		X
197	Diclofop_methyl		X
198	Dicloran		X
199	Dicofol	O	
200	Dieldrin	O	
201	Diethatyl-ethyl		X
202	Diethofencarb	O	
203	Difenoconazole	O	
204	Diflufenican		X
205	Dimepiperate		X
206	Dimethachlor		X
207	Dimethametryn	O	
208	Dimethenamid	O	
209	Dimethoate	O	
210	Dimethylvinphos		X
211	Diniconazole	O	
212	Dinitramine		X
213	Dioxathion		X
214	Diphenamid		X
215	Diphenylamine	O	
216	Dithiopyr	O	
217	Edifenphos	O	
218	Endosulfan	O	
219	Flamprop-isopropyl		X
220	Flonicamid	O	

221	Fluazifop_butyl	O	
222	Fluchloralin		X
223	Flucythrinate		X
224	Fludioxonil	O	
225	Flufenpyr_ethyl		X
226	Flumetralin		X
227	Flumiclorac_pentyl		X
228	Flumioxazine	O	
229	Fluopyram	O	
230	Fluorodifen		X
231	Flurochloridone		X
232	Flusilazole	O	
233	Flutamone		X
234	Fluthiacet_methyl	O	
235	Flutianil	O	
236	Flutolanil	O	
237	Flutriafol	O	
238	Fluvalinate		X
239	Folpet	O	
240	Fonofos		X
241	Formothion		X
242	Fosthiazate	O	
243	Fthalide	O	
244	Furathiocarb	O	
245	Halfenprox		X
246	Heptachlor	O	
247	Heptachlor-epoxide	O	
248	Heptenophos		X
249	Hexachlorbenzene		X
250	Hexaconazole	O	
251	Molinate	O	
252	Monolinuron		X
253	Myclobutanil	O	
254	Napropamide	O	
255	Nitrapyrin	O	
256	Nitrothal-isopropyl		X
257	Nonachlor_cis		X

258	Nonachlor_trans		X
259	Norflurazon		X
260	Nuarimol		X
261	Ofurace	O	
262	Oxadiazon	O	
263	Oxadixyl	O	
264	Oxyflofen	O	
265	Paclobutrazole	O	
266	Parathion-ethyl		X
267	Parathion-methyl	O	
268	Pebulate		X
269	Penconazole	O	
270	Pendimethalin	O	
271	Penflufen	O	
272	Pentachlorobezonitrile		X
273	Penthiopyrad	O	
274	Pentoxazon	O	
275	Permethrin	O	
276	Perthane		X
277	Phenothrin		X
278	Phenthoate	O	
279	Phorate	O	
280	Phosalone	O	
281	Phosmet(PMP)	O	
282	Phosphamidone		X
283	Picolinafen		X
284	Picoxystrobin	O	
285	Quintozene	O	
286	Sectumeton		X
287	Silafluofen	O	
288	Simeconazole	O	
289	Simetryn	O	
290	Spiromesifen	O	
291	Spiroxamine	O	
292	Sulfotep		X
293	Sulprofos		X
294	TCMTB		X

295	Tebuconazole	O	
296	Tebufenpyrad	O	
297	Tebupirimfos	O	
298	Tefluthrin	O	
299	Terbacil		X
300	Terbufos	O	
301	Terbumeton		X
302	Terbuthylazine		X
303	Terbutryn		X
304	Tetrachlorvinphos		X
305	Tetraconazole	O	
306	Tetradifon	O	
307	Tetramethrin		X
308	Tetrasul		X
309	Thiazopyr		X
310	Thifluzamide	O	
311	Thiometon		X
312	Thionazin		X
313	Tolclofos_methyl	O	
314	Tolfenpyrad	O	
315	Tolylfluanid		X
316	Tralomethrin	O	
317	Triflumuron	O	
318	Trifluralin	O	
319	Uniconazole		X
320	Vernlolate		X
321	Vinclozoline	O	
322	Zoxamide	O	

Table S2. Average recovery(%), Limit of quantification(ng/g), SD(%), RSD(%) and matrix effect for the GC-MSMS method applied to coffee.

	Coffee					
Compound	Average recovery	LOQ	LOD	SD	RSD	Matrix Effect
	(%)	(ng/g)	(ng/g)	(%)	(%)	(%)
2,6-Diisoporpylnaphthalene	69.1	4.1	1.15	8.6	12.45	20
Acetochlor	75.9	0.1	0.03	5.1	6.72	5
Acibenzola_s_methyl	92.4	3	0.66	12.3	13.31	13
Acrinathrin_1	86.5	0.85	0.26	3.8	4.39	-15
Acrinathrin_2	81.5	0.83	0.22	3.7	4.54	-17
Alachlor	79.2	0.2	0.05	5.8	7.32	15
Aldrin	65.2	0.1	0.02	7.6	11.66	21
Allethrin-1	60.5	1.32	0.33	16.6	27.44	8
Allethrin-2	83.8	1.32	0.26	10.0	11.93	13
Allidochlor	72.3	0.03	0.01	4.2	5.81	-15
Ametryn	78.8	0.01	0.00	9.7	12.31	18
Anilofos	76.2	0.1	0.03	5.9	7.74	24
Aramit-1	92.7	0.19	0.05	3.0	3.24	24
Aramit-2	66.4	0.19	0.04	1.4	2.11	10
Aspon	77.7	0.09	0.02	8.4	10.81	-14
Atrazine	73.5	0.2	0.06	8.7	11.84	-6
Azaconazole	76.5	0.21	0.05	9.2	12.03	-13
Azinphos-ethyl	75.6	1.15	0.25	4.7	6.22	28
Azinphos-methyl	93.5	0.7	0.20	3.4	3.64	-8
Benalaxyl	82.6	0.17	0.04	7.8	9.44	20
Benodanil	84.7	0.13	0.03	9.6	11.33	-20
Benoxacor	125.6	0.29	0.06	56	44.59	12
Benzoylprop_ethyl	82.9	0.39	0.09	6.5	7.84	5
BHC_alpha	69.5	0.17	0.04	5.6	8.06	55
BHC_beta	71.6	0.17	0.04	6	8.38	-11
BHC_delta	69.8	0.17	0.04	6.2	8.88	14
BHC_gamma	71.9	0.17	0.04	4.9	6.82	4
Bifenox	86.6	0.21	0.05	3.6	4.16	-6
Bifenthrin	77.5	0.13	0.03	8.6	11.10	-10
Bromacil	74.4	1	0.26	11.3	15.19	9
Bromobutide	81.5	1.5	0.39	6.9	8.47	26
Bromophos-ethyl	74.7	0.15	0.04	9.4	12.58	29
Bromophos-methyl	76.4	0.2	0.06	7.4	9.69	13
Bromopropylate	80.1	0.21	0.05	7.4	9.24	15

Bupirimate	81.7	0.16	0.04	5.6	6.85	-14
Butachlor	83.9	0.89	0.19	10.6	12.63	1
Butafenacil	82.5	0.38	0.08	4.8	5.82	16
Butralin	84.2	0.21	0.06	7.4	8.79	6
Butylate	69	0.4	0.08	5.3	7.68	5
Cadusafos	77.7	0.15	0.03	4	5.15	2
Captan	79.8	5.5	1.27	3.2	4.01	24
Carbophenothion	78.5	0.1	0.03	11.6	14.78	-2
Chinomethionat	71.3	0.21	0.04	7.6	10.66	-16
Chlorbenside	70.2	0.17	0.04	11.4	16.24	18
Chlorbufam	69	1.3	0.34	8.3	12.03	-2
Chlordane_1	73.5	0.31	0.09	11.1	15.10	-16
Chlordane_2	70.1	0.31	0.08	11.4	16.26	25
Chlorethoxyfos	72.7	0.73	0.15	3.9	5.36	14
Chlorfenapyr	75.8	1	0.21	4.2	5.54	8
Chlorfenson	77.1	0.14	0.04	9.1	11.80	50
Chlorfluazuron	26.4	0.3	0.08	12.1	45.83	16
Chlorflurenol_methyl	80.4	0.21	0.05	7.3	9.08	14
Chlornitrofen	87.5	0.23	0.05	5.7	6.51	26
Chlorobenzilate	82.9	0.15	0.03	7	8.44	16
Chloroneb	72.6	1.69	0.46	6	8.26	12
Chloropropylate	80.2	0.11	0.03	8.2	10.22	5
Chloroxuron	65.5	0.33	0.09	4.7	7.18	30
Chlorpropham	74.8	0.24	0.05	7.8	10.43	-2
Chlorpyrifos	82.2	0.14	0.04	6.3	7.66	29
Chlorpyrifos-methyl	76.4	0.11	0.02	6.7	8.77	19
Chlorthal-dimethyl	83.6	0.07	0.02	6.4	7.66	-16
Chlorthalonil	77.2	2.8	0.78	11.4	14.77	-5
Chlorthion	83.1	0.23	0.07	5.8	6.98	17
Chlorthiophos_1	74.2	0.22	0.05	9.5	12.80	18
Chlorthiophos_2	74.7	0.22	0.05	9.2	12.32	30
Chlozolinate	80.5	0.3	0.06	6.8	8.45	-15
Cinidone_ethyl	84	0.35	0.08	8.3	9.88	-7
Cinmethylin	83.2	1.7	0.34	8.3	9.98	29
Clomazon	79.4	0.09	0.02	8.2	10.33	59
Clomeporp	82.6	0.13	0.04	3.4	4.12	6
Coumaphos	75.1	3.5	0.88	4.8	6.39	5
Crotoxyphos	80.2	0.15	0.05	8.5	10.60	-16
Cyanazine	79.4	0.17	0.04	8.9	11.21	7
Cyanophos	79.3	0.07	0.02	4.6	5.80	3
Cycloate	72.4	0.03	0.01	5.2	7.18	48
Cyflufenamid	82.6	0.15	0.03	8.9	10.77	2

Cyfluthrin-1	76.2	1	0.20	7.3	9.58	7
Cyfluthrin-2	78.5	1	0.29	6.5	8.28	22
Cyfluthrin-3	77	1	0.22	7.7	10.00	20
Cyfluthrin-4	80.3	1	0.22	6.7	8.34	-16
Cyhalofop-butyl	79.6	0.8	0.21	5.9	7.41	13
Cyhalothrin-1	82	0.9	0.23	4.7	5.73	-17
Cyhalothrin-2	86.3	0.9	0.26	1.7	1.97	27
Cypermethrin-1	79.9	1.1	0.31	7.3	9.14	10
Cypermethrin-2	80.4	1.1	0.30	6.9	8.58	9
Cypermethrin-3	82.1	1.1	0.30	2.8	3.41	14
Cypermethrin-4	81.6	1.1	0.28	7.1	8.70	-5
Cyprazine	79.8	0.17	0.04	6.6	8.27	-2
Cyproconazole-1	79.3	0.6	0.14	8.2	10.34	27
Cyproconazole-2	79.2	0.6	0.17	7.2	9.09	22
Cyprodinil	79.6	0.1	0.02	8.4	10.55	9
DDD_pp	72.3	0.1	0.03	7.7	10.65	23
DDE_pp	62.3	4.5	1.13	8.8	14.13	30
DDT_op	65.2	0.1	0.02	8.9	13.65	49
DDT_pp	66.8	0.1	0.03	8.5	12.72	11
Deltamethrin-1	116.4	0.1	0.03	6.4	5.50	15
Deltamethrin-2	80.3	0.1	0.02	6.3	7.85	21
Demeton_O	76.3	0.26	0.08	4.2	5.50	25
Demeton_S	71.9	0.13	0.03	7.7	10.71	21
Demeton_S_methylsulfone	23.8	1.33	0.37	20	84.03	-18
Desmetryn	76.5	8.6	1.89	5.9	7.71	17
Diafor	78.8	0.9	0.19	1.9	2.41	24
Diallate-1	74	0.11	0.03	8.2	11.08	16
Diallate-2	71.7	0.11	0.03	7.3	10.18	9
Diazinon	74.6	0.21	0.06	7.5	10.05	25
Dichlofenthion	75.3	0.16	0.04	5.6	7.44	21
Dichlofluanid	80.5	7.6	1.98	7.5	9.32	55
Dichlormid	74.4	0.36	0.10	7.4	9.95	6
Dichlorvos	62.7	7.5	1.58	9.4	14.99	26
Diclobutrazole	83.7	0.4	0.11	6.4	7.65	-5
Diclofop_methyl	79.1	0.21	0.05	8.6	10.87	2
Dicloran	73	0.31	0.09	6	8.22	5
Dicofol	73.8	0.15	0.03	8	10.84	-17
Dieldrin	73.6	0.51	0.14	5.7	7.74	-15
Diethatyl-ethyl	83.5	0.13	0.03	8.9	10.66	-4
Diethofencarb	79.4	0.21	0.06	9.3	11.71	7
Difenoconazole-1	80.7	0.36	0.10	5.6	6.94	29
Difenoconazole-2	77.7	0.36	0.10	4.8	6.18	5

Diflufenican	80.7	0.26	0.07	6.6	8.18	36
Dimepiperate	77.6	2.1	0.57	8.3	10.70	3
Dimethachlor	78.2	0.6	0.16	6.3	8.06	-7
Dimethametryn	79.5	0.16	0.04	8.9	11.19	-6
Dimethenamid	79.6	0.16	0.04	7.4	9.30	-17
Dimethoate	78.1	1.2	0.29	7.6	9.73	5
Dimethylvinphos-(E)	79.4	0.13	0.04	6	7.56	12
Dimethylvinphos-(Z)	79.6	0.13	0.04	5.6	7.04	-16
Diniconazole	78.6	0.31	0.07	4.7	5.98	20
Dinitramine	79	0.61	0.17	6	7.59	14
Dioxathion	80.4	4.6	0.97	6.2	7.71	-9
Diphenamid	79.8	0.17	0.05	7.7	9.65	8
Diphenylamine	75.8	0.21	0.05	5.9	7.78	-5
Dithiopyr	82	0.17	0.04	7.8	9.51	6
Edifenphos	73.7	0.12	0.04	5.6	7.60	-3
Endosulfan_alpha	213.1	0.56	0.13	26.4	12.39	8
Endosulfan_beta	77.7	0.41	0.10	9.3	11.97	-1
Endosulfan_sulfate	73.2	0.31	0.09	8.7	11.89	-18
Endrin	75.8	0.31	0.07	6.9	9.10	21
EPN	81.1	0.57	0.11	6.4	7.89	1
Epoxiconazole	79.7	0.2	0.05	6.6	8.28	-19
EPTC	67	0.37	0.08	5.5	8.21	22
Esprocarb	77.8	0.1	0.03	9.2	11.83	15
Etaconazole_1	84.6	0.26	0.08	7.2	8.51	2
Etaconazole_2	79.1	0.26	0.08	6.3	7.96	28
Ethalfluralin	77.9	0.1	0.02	2.4	3.08	-7
Ethion	82	0.21	0.06	7.8	9.51	-10
Ethofumesate	87	0.2	0.05	9.5	10.92	14
Ethoprophos	77	0.2	0.05	6.9	8.96	-4
Ethychlozate	83.9	0.3	0.09	11	13.11	16
Etofenprox	72.6	6.8	1.90	7.5	10.33	-12
Etoxazole	114.2	1.2	0.29	14.7	12.87	23
Etridiazole	71.8	0.5	0.13	4.8	6.69	11
Etrimfos	75.1	0.1	0.02	8.1	10.79	19
Fenamidone	82.2	0.1	0.03	5.6	6.81	6
Fenamiphos	80	0.2	0.04	12.7	15.88	14
Fenarimol	78.8	0.21	0.04	4.6	5.84	12
Fenazaquin	69.3	0.5	0.15	7.1	10.25	-16
Fenbuconazole	76.2	1.6	0.48	5.8	7.61	9
Fenchlorphos	75.3	0.23	0.06	5.7	7.57	17
Fenclorim	74.9	0.1	0.03	8.8	11.75	16
Fenfuram	79.8	0.21	0.06	7.2	9.02	2

Fenitrothion	110.3	0.31	0.08	5.5	4.99	-13
Fenobucarb	75.9	2.6	0.70	5.9	7.77	24
Fenothiocarb	78.9	1	0.27	6.6	8.37	-8
Fenoxanil	82	0.13	0.03	8	9.76	-12
Fenoxycarb	57.6	0.6	0.17	9.7	16.84	7
Fenpropathrin	82.7	0.1	0.02	7.7	9.31	13
Fenson	75.5	0.23	0.06	7.2	9.54	24
Fenthion	80.5	0.15	0.04	7.9	9.81	10
Fenvalerate-1	75.1	0.21	0.05	8.1	10.79	20
Fenvalerate-2	83	0.21	0.06	8.4	10.12	16
Fipronil	84.1	0.1	0.03	5.2	6.18	30
Flamprop-isopropyl	86.1	0.23	0.06	7.1	8.25	22
Flonicamid	55.8	0.2	0.05	21.8	39.07	16
Fluazifop_butyl	81.6	0.25	0.06	9.2	11.27	-8
Fluchloralin	77.8	0.1	0.02	3.7	4.76	1
Flucythrinate_1	80.9	0.36	0.07	5.6	6.92	19
Flucythrinate_2	81.6	0.36	0.08	5	6.13	27
Fludioxonil	83.4	0.1	0.02	11	13.19	25
Flufenpyr_ethyl	77.9	0.35	0.09	8.8	11.30	-3
Flumetralin	86.9	0.28	0.08	6.4	7.36	-1
Flumiclorac_pentyl	77.9	0.37	0.10	8.8	11.30	-16
Flumioxazine	87.9	0.21	0.05	5.1	5.80	22
Fluopyram	82.1	0.13	0.03	10	12.18	9
Fluorodifen	84.2	0.27	0.07	8.7	10.33	-12
Flurochloridone	80.7	0.24	0.06	8.8	10.90	24
Flusilazole	79.6	0.1	0.02	7.8	9.80	12
Flutamone	82.6	0.56	0.16	4.8	5.81	-18
Fluthiacet_methyl	87.2	0.02	0.01	10.2	11.70	-14
Flutianil	86.1	0.2	0.04	1.6	1.86	21
Flutolanil	78.6	0.1	0.02	9.1	11.58	18
Flutriafol	88.9	0.03	0.01	17.3	19.46	9
Fluvalinate-1	80.1	0.64	0.18	7	8.74	20
Fluvalinate-2	82.7	0.64	0.16	6.2	7.50	-6
Folpet	79.8	4.3	1.08	8.5	10.65	10
Fonofos	76.1	2.3	0.48	6.8	8.94	-11
Formothion	76.4	0.1	0.02	12.9	16.88	-1
Fosthiazate-1	80.5	0.37	0.10	7.4	9.19	13
Fosthiazate-2	76.1	0.37	0.08	9.6	12.61	-15
Fthalide	77.8	0.1	0.03	7.6	9.77	14
Furathiocarb	81.9	0.1	0.02	3.3	4.03	24
Halfenprox	76	0.9	0.18	7.4	9.74	-7
Heptachlor	67.6	0.3	0.06	5.5	8.14	-6

Heptachlor-epoxide	64.4	0.14	0.03	8.6	13.35	13
Heptenophos	76.4	0.33	0.07	6	7.85	-9
Hexachlorbenzene	62.8	0.1	0.03	10.7	17.04	11
Hexaconazole	74.1	0.1	0.02	5.8	7.83	17
Imazalil	32.6	0.1	0.03	28.4	87.12	22
Indanofan	80.1	0.33	0.08	6.6	8.24	-9
Indoxacarb	82.6	3	0.81	2.8	3.39	22
Iprobenfos	79.5	0.16	0.03	4.1	5.16	21
Iprodione	77.5	0.1	0.03	8.8	11.35	-16
Iprovalicarb-1	80.7	2.6	0.52	8	9.91	6
Iprovalicarb-2	79.9	2.6	0.75	9.6	12.02	17
Isazofos	80.2	0.41	0.11	6.7	8.35	-19
Isofenphos	82.3	0.16	0.04	6.7	8.14	13
Isofenphos-methyl	82.1	0.09	0.02	7	8.53	-5
Isopropalin	79.1	0.16	0.03	6.4	8.09	49
Isoprothiolane	81.5	0.13	0.03	6.1	7.48	14
Isotianil	86.4	0.76	0.19	8.5	9.84	56
Isoxadifen-ethyl	82.4	0.34	0.07	7.8	9.47	20
Isoxanthion	83.9	0.008	0.00	9.5	11.32	-9
Kresoxim-methyl	83.3	0.36	0.10	9.1	10.92	-19
Lactofen	85.3	0.73	0.22	2.2	2.58	-19
Leptophos	67.9	0.2	0.05	9.1	13.40	10
Malathion	82	0.5	0.11	11.3	13.78	25
Mecarbam	84.3	1.5	0.30	6	7.12	12
Mefenacet	77.1	0.1	0.03	7.5	9.73	59
Mefenpyr-diethyl	82	0.27	0.06	6.1	7.44	4
Mepronil	82	0.32	0.08	10	12.20	9
Metazachlor	84.6	0.1	0.02	8	9.46	25
Metconazole	78.7	1.1	0.22	5.3	6.73	17
Methidathion	80.6	0.14	0.03	7.5	9.31	28
Methoprotryne	80.4	0.2	0.06	9.4	11.69	-14
Methoxychlor	71.9	1.5	0.32	7.6	10.57	-16
Methyltrithion	82.3	0.32	0.07	9.3	11.30	22
Metolachlor	78.6	0.25	0.06	7	8.91	28
Metrafenone	74.9	0.29	0.08	7.9	10.55	5
Metribuzin	77.1	0.16	0.04	4	5.19	2
MGK-264_1	78.6	0.4	0.12	7.1	9.03	21
MGK-264_2	80.1	0.4	0.10	9.5	11.86	8
Mirex	53.6	0.09	0.02	9.6	17.91	11
Molinate	71.8	2.5	0.53	6.6	9.19	-15
Monolinuron	69.8	0.3	0.08	2.5	3.58	-19
Myclobutanil	79.9	0.13	0.04	9.6	12.02	2

Napropamide	84.5	0.6	0.14	13.3	15.74	27
Nitrapyrin	71.8	0.41	0.12	2.8	3.90	-4
Nitrothal-isopropyl	85.5	0.4	0.08	3.1	3.63	-11
Nonachlor_cis	66.3	0.15	0.03	9.8	14.78	7
Nonachlor_trans	61.7	0.15	0.04	11.1	17.99	16
Norflurazon	82.4	0.31	0.09	7.9	9.59	24
Nuarimol	80	0.35	0.08	7.8	9.75	25
Ofurace	77.2	0.2	0.04	7.3	9.46	-19
Oxadiazon	79.3	0.3	0.07	8.8	11.10	6
Oxadixyl	56.6	0.17	0.04	22.3	39.40	17
Oxyflofen	86.9	0.39	0.09	3.7	4.26	7
Paclobutrazole	81.2	0.42	0.12	8.4	10.34	26
Parathion-ethyl	85.6	0.1	0.03	4.8	5.61	25
Parathion-methyl	79.7	0.33	0.08	2.1	2.63	15
Pebulate	68.6	0.009	0.00	6.1	8.89	25
Penconazole	83.2	0.15	0.03	8.3	9.98	6
Pendimethalin	80.6	0.26	0.07	5.3	6.58	8
Penflufen	82.3	0.22	0.06	7.1	8.63	14
Pentachlorobezonitrile	79.4	0.27	0.08	1.7	2.14	26
Penthiopyrad	81.4	0.23	0.06	8.9	10.93	5
Pentoxazon	78	0.38	0.08	5.9	7.56	-3
Permethrin-1	71.9	0.61	0.15	8.8	12.24	15
Permethrin-2	130.3	0.61	0.15	8.5	6.52	-6
Perthane	69.6	0.16	0.05	7.8	11.21	-2
Phenothrin	97.5	0.003	0.00	7.8	8.00	-16
Phenthoate	80.8	0.38	0.10	7.2	8.91	-3
Phorate	75.2	0.1	0.03	5.2	6.91	17
Phosalone	76.7	0.51	0.12	7.5	9.78	-13
Phosmet(PMP)	83.6	0.91	0.23	7.3	8.73	-10
Phosphamidone	85.1	0.2	0.05	17.3	20.33	28
Picolinafen	80.7	0.16	0.04	6.9	8.55	18
Picoxystrobin	85.7	0.12	0.03	8.5	9.92	18
Piperonyl butoxide	79.9	0.25	0.07	8.9	11.14	-4
Piperophos	80.9	1.2	0.30	6	7.42	26
Pirimiphos-ethyl	78.1	0.19	0.05	10.2	13.06	18
Pirimiphos-methyl	82.7	0.19	0.06	7.3	8.83	9
Pretilachlor	81.2	0.16	0.04	8.6	10.59	22
Primicarb	78.2	0.18	0.05	6.6	8.44	-7
Probenazole	83.3	0.4	0.11	7.4	8.88	7
Prochloraz	75.9	1.2	0.25	7	9.22	22
Procymidone	83.6	1.3	0.39	8	9.57	-4
Profenofos	74.1	0.3	0.08	8.6	11.61	-5

Profluralin	81.4	0.43	0.09	1.3	1.60	13
Prometon	75.2	0.65	0.15	4.7	6.25	25
Prometryn	77.5	0.3	0.08	8.3	10.71	-16
Pronamide	79.5	0.006	0.00	3.5	4.40	-16
Propachlor	75	0.12	0.03	6.3	8.40	11
Propanil	83.4	0.17	0.04	7.8	9.35	13
Propazine	74.9	0.86	0.23	9.1	12.15	10
Propetamphos	77.8	0.52	0.13	4.2	5.40	20
Propham	72.7	0.64	0.13	14.2	19.53	-4
Propiconazole-1	80.5	0.51	0.14	6	7.45	28
Propiconazole-2	77.8	0.51	0.14	6.7	8.61	12
Propisochlor	80.4	0.8	0.22	5.7	7.09	24
Prothiophos	76.3	0.21	0.06	7.9	10.35	9
Pyracabolid	85.7	9.3	2.70	8.6	10.04	-26
Pyraclofos	72.4	0.22	0.04	6.9	9.53	6
Pyrazophos	77.9	0.72	0.19	3.4	4.36	20
Pyridaben	119.5	0.26	0.08	6	5.02	22
Pyridalyl	70.2	0.19	0.05	8.4	11.97	-5
Pyridaphenthion	84.1	0.38	0.11	6.1	7.25	19
Pyrifenox 1	76.4	0.89	0.24	6.7	8.77	48
Pyrifenox 2	79.9	5.61	1.57	9.3	11.64	-17
Pyrimidifen	81.7	0.21	0.05	4.2	5.14	-14
Pyriminobac-methyl(E)	79.8	0.7	0.14	7.5	9.40	24
Quinalphos	82.3	0.83	0.18	5.7	6.93	-15
Quinoxyfen	75.8	0.21	0.06	8.5	11.21	25
Quintozene	71.5	0.31	0.08	4.1	5.73	38
Sectumeton	79.8	0.29	0.08	11.9	14.91	23
Silafluofen	69.1	0.14	0.04	8	11.58	-26
Simeconazole	76.2	0.16	0.04	5.8	7.61	24
Simetryn	93.6	0.4	0.09	5.5	5.88	18
Spiromesifen	76.6	0.18	0.04	8.7	11.36	-17
Spiroxamine 1	68.8	1.11	0.26	4.3	6.25	12
Spiroxamine_2	70.2	1.11	0.22	6.4	9.12	-15
Sulfotep	73.6	0.2	0.04	5.9	8.02	55
Sulprofos	76.4	0.008	0.00	7.6	9.95	14
TCMTB	88.1	0.02	0.01	12.7	14.42	-13
Tebuconazole	77.7	0.31	0.08	7.4	9.52	4
Tebufenpyrad	79.3	0.21	0.04	4.4	5.55	5
Tebupirimfos	76.4	0.16	0.05	5.8	7.59	13
Tefluthrin	76	0.13	0.03	8	10.53	46
Terbacil	79.6	0.85	0.23	7.3	9.17	-17
Terbufos	73.5	0.4	0.09	6.4	8.71	-18

Terbumeton	74.2	0.63	0.19	9.3	12.53	15
Terbuthylazine	74.5	0.23	0.06	4.9	6.58	-20
Terbutryn	77.9	0.31	0.07	8.5	10.91	29
Tetrachlorvinphos	76.8	0.13	0.04	5.5	7.16	18
Tetraconazole	81.3	0.06	0.01	9.1	11.19	10
Tetradifon	79.9	0.17	0.04	6.9	8.64	-17
Tetramethrin-1	82.2	0.89	0.26	7.3	8.88	24
Tetramethrin-2	84	0.89	0.20	6.7	7.98	5
Tetrasul	62.7	0.06	0.01	10.9	17.38	8
Thiazopyr	83.2	1.6	0.37	7	8.41	29
Thifluzamide	86.1	0.31	0.07	8.6	9.99	10
Thiometon	71.2	0.26	0.07	4.4	6.18	-14
Thionazin	76.5	0.33	0.07	2.6	3.40	-26
Tolclofos_methyl	76.3	0.28	0.06	6.3	8.26	21
Tolfenpyrad	77.9	0.26	0.05	6.4	8.22	47
Tolylfluanid	81	5.1	1.07	7.5	9.26	28
Tralomethrin-1	116.4	1.6	0.34	2.6	2.23	-32
Tralomethrin-2	78.8	1.6	0.42	2.8	3.55	-15
Triadimefon	76.5	0.21	0.05	9.1	11.90	-12
Triadimenol	80	1.5	0.44	7.9	9.88	9
Triallate	72.1	0.22	0.05	9.6	13.31	17
Triazophos	83.8	2.73	0.68	8.8	10.50	49
Tribufos	79.5	0.005	0.00	7.8	9.81	9
Tridiphane	70.7	0.62	0.15	8.3	11.74	12
Triflumizole	79.6	0.52	0.12	7.3	9.17	42
Triflumuron	82.4	0.4	0.09	4.8	5.83	16
Trifluralin	77.4	0.13	0.03	3.7	4.78	16
Uniconazole	80.5	0.57	0.13	7.5	9.32	28
Vernlolate	68.6	0.006	0.00	6.8	9.91	-7
Vinclozoline	78.5	0.21	0.06	4.6	5.86	26
Zoxamide	95.4	0.06	0.02	11.2	11.74	48

LOD: Limit of Dtection
LOQ: Limit of Quantification
SD: Standard Deviation
RSD: Relative Standard Deviation

Table S3. Average recovery(%), Limit of quantification(ng/g), SD(%), RSD(%) and matrix effect for the GC-MSMS method applied to potato.

	potato					
Compound	Average recovery	LOQ	LOD	SD	RSD	Matrix Effect
	(%)	(ng/g)	(ng/g)	(%)	(%)	(%)
2,6-Diisoporpylnaphthalene	72.4	4.1	1.19	2.4	3.31	6
Acetochlor	74.3	0.1	0.03	4.8	6.46	22
Acibenzola_s_methyl	66.4	4.5	1.35	1.6	2.41	-6
Acrinathrin_1	77.6	0.61	0.13	4.0	5.15	6
Acrinathrin_2	77.2	0.65	0.20	8.1	10.49	15
Alachlor	73.7	0.2	0.05	2.1	2.85	33
Aldrin	63.8	0.1	0.03	1.0	1.57	26
Allethrin-1	86.1	1.25	0.26	5.9	6.85	4
Allethrin-2	77.3	1.25	0.31	5.4	6.99	-10
Allidochlor	61.2	0.03	0.01	5.0	8.17	-15
Ametryn	72.4	0.01	0.00	3.7	5.11	-2
Anilofos	70.7	0.08	0.02	3.2	4.53	26
Aramit-1	89.1	0.19	0.05	9.3	10.44	29
Aramit-2	73.9	0.19	0.05	2.4	3.25	17
Aspon	73.1	0.09	0.02	2.6	3.56	15
Atrazine	69.7	0.2	0.05	2.3	3.30	39
Azaconazole	70.7	0.19	0.06	2.1	2.97	-10
Azinphos-ethyl	72.6	1.1	0.25	4.8	6.61	-11
Azinphos-methyl	67.8	0.09	0.02	6.4	9.44	-19
Benalaxyl	77.4	0.19	0.05	1.7	2.20	-2
Benodanil	72.7	0.12	0.03	3.4	4.68	-2
Benoxacor	70.7	0.5	0.11	2.3	3.25	47
Benzoylprop_ethyl	76.9	0.33	0.07	2.2	2.86	21
BHC_alpha	67.7	0.17	0.04	2.5	3.69	20
BHC_beta	70.1	0.17	0.04	1.3	1.85	29
BHC_delta	70.2	0.17	0.04	2.7	3.85	-17
BHC_gamma	67.9	0.17	0.04	2	2.95	-6
Bifenox	61.5	0.21	0.04	3.6	5.85	28
Bifenthrin	76.5	0.13	0.03	2.5	3.27	-19
Bromacil	75.1	0.9	0.22	1.7	2.26	9
Bromobutide	72.8	1	0.26	2.9	3.98	55
Bromophos-ethyl	70.6	0.16	0.04	3	4.25	25
Bromophos-methyl	71.6	0.2	0.06	4.5	6.28	8
Bromopropylate	76.7	0.21	0.06	4	5.22	-15

Bupirimate	71.6	0.16	0.04	3.6	5.03	29
Butachlor	77	0.5	0.12	1.3	1.69	8
Butafenacil	76.9	0.36	0.08	5.4	7.02	10
Butralin	71.4	0.2	0.04	2.4	3.36	-12
Butylate	58.6	0.45	0.12	1.4	2.39	4
Cadusafos	72	0.16	0.03	3.6	5.00	50
Captan	78.1	4.5	1.04	7.4	9.48	2
Carbophenothion	71.6	0.1	0.02	3	4.19	33
Chinomethionat	68.7	0.21	0.05	2.5	3.64	2
Chlorbenside	64.5	0.17	0.04	5.2	8.06	4
Chlorbufam	71.9	1.25	0.29	4.3	5.98	-10
Chlordane_1	71.7	0.32	0.08	1.5	2.09	26
Chlordane_2	68	0.32	0.09	1.2	1.76	23
Chlorethoxyfos	67.9	0.8	0.22	2.6	3.83	-18
Chlorfenapyr	73	1.2	0.24	4.6	6.30	48
Chlorfenson	72.9	0.15	0.03	3	4.12	-17
Chlorfluazuron	76.2	0.2	0.05	11.9	15.62	16
Chlorflurenol_methyl	70.6	0.25	0.07	2.6	3.68	45
Chlornitrofen	69.1	0.23	0.06	4.1	5.93	-8
Chlorobenzilate	72.5	0.15	0.03	2.9	4.00	-9
Chloroneb	67.8	1.7	0.37	2.4	3.54	13
Chloropropylate	71.5	0.16	0.04	3.1	4.34	55
Chloroxuron	70.5	0.36	0.09	3.3	4.68	27
Chlorpropham	76	0.26	0.07	1.9	2.50	21
Chlorpyrifos	74.8	0.31	0.06	3.4	4.55	19
Chlorpyrifos-methyl	71.2	0.11	0.03	1.6	2.25	16
Chlorthal-dimethyl	76.3	0.06	0.01	3.4	4.46	-8
Chlorthalonil	83.1	2.9	0.67	2.2	2.65	-19
Chlorthion	66.8	0.25	0.07	4.3	6.44	-14
Chlorthiophos_1	72.1	0.22	0.06	2.6	3.61	27
Chlorthiophos_2	73.4	0.22	0.05	2.6	3.54	21
Chlozolinate	72.1	0.31	0.07	2.6	3.61	-13
Cinidone_ethyl	79.3	0.31	0.06	3.9	4.92	59
Cinmethylin	82.3	1.7	0.37	3.4	4.13	16
Clomazon	72.5	0.08	0.02	3.8	5.24	11
Clomeporp	71.6	0.13	0.03	1.6	2.23	18
Coumaphos	72.9	3.45	1.04	4.1	5.62	7
Crotoxyphos	72.5	0.145	0.04	5.6	7.72	-12
Cyanazine	72.8	0.18	0.05	3.1	4.26	26
Cyanophos	72.6	0.07	0.02	3.3	4.55	-20
Cycloate	67.5	0.05	0.01	2.2	3.26	17
Cyflufenamid	76.4	2.3	0.62	3.7	4.84	38

Cyfluthrin-1	78.6	1.1	0.23	4.5	5.73	-12
Cyfluthrin-2	78.2	1.1	0.22	4.3	5.50	8
Cyfluthrin-3	78.2	1.1	0.32	2.7	3.45	17
Cyfluthrin-4	77.4	1.1	0.24	4.4	5.68	24
Cyhalofop-butyl	77.9	0.8	0.18	2.9	3.72	19
Cyhalothrin-1	78.4	0.6	0.16	3.5	4.46	26
Cyhalothrin-2	74.5	0.6	0.16	2.5	3.36	50
Cypermethrin-1	80.1	1.1	0.32	4.4	5.49	21
Cypermethrin-2	79.4	1.1	0.31	3.9	4.91	25
Cypermethrin-3	79.3	1.1	0.30	4.9	6.18	43
Cypermethrin-4	75.9	1.1	0.30	2.7	3.56	17
Cyprazine	72.3	0.17	0.04	2.2	3.04	24
Cyproconazole-1	72.4	0.5	0.11	2.9	4.01	26
Cyproconazole-2	71.8	0.5	0.12	1.9	2.65	13
Cyprodinil	73.3	0.1	0.03	2.5	3.41	28
DDD_pp	67.6	0.1	0.02	2.6	3.85	-15
DDE_pp	69.3	3.9	1.05	2.8	4.04	-20
DDT_op	68.5	0.1	0.03	1.9	2.77	24
DDT_pp	70.2	0.1	0.02	1.9	2.71	21
Deltamethrin-1	67.6	0.1	0.03	6.1	9.02	-7
Deltamethrin-2	77.4	0.1	0.03	4.4	5.68	6
Demeton_O	53.8	0.25	0.06	8	14.87	21
Demeton_S	49.8	0.15	0.04	5.4	10.84	26
Demeton_S_methylsulfone	6.8	1.32	0.30	4	58.82	-16
Desmetryn	69.3	7.9	2.21	2.5	3.61	20
Diafor	75.2	0.87	0.19	5.1	6.78	26
Diallate-1	68.5	0.12	0.03	2.7	3.94	27
Diallate-2	68.9	0.12	0.03	3.8	5.52	19
Diazinon	74.6	0.21	0.06	1.6	2.14	-17
Dichlofenthion	71.6	0.15	0.04	3.9	5.45	-18
Dichlofluanid	78.6	7	1.61	4.1	5.22	5
Dichlormid	61.5	0.4	0.10	3.7	6.02	3
Dichlorvos	62.1	7.8	2.18	7.4	11.92	20
Diclobutrazole	69.8	0.36	0.08	3.2	4.58	36
Diclofop_methyl	78.2	0.221	0.06	2.2	2.81	-3
Dicloran	68.8	0.321	0.08	3.2	4.65	16
Dicofol	71.1	0.14	0.04	2.5	3.52	24
Dieldrin	68.2	0.51	0.11	6.1	8.94	42
Diethatyl-ethyl	73.8	0.15	0.04	2.6	3.52	-11
Diethofencarb	75.1	0.2	0.04	2.5	3.33	16
Difenoconazole-1	72.8	0.31	0.09	3.4	4.67	8
Difenoconazole-2	71.9	0.31	0.09	2.5	3.48	13

Diflufenican	75.9	0.21	0.06	1.7	2.24	-16
Dimepiperate	72	2.91	0.81	1.9	2.64	9
Dimethachlor	73.8	0.51	0.14	3.2	4.34	24
Dimethametryn	72.3	0.12	0.03	2.9	4.01	46
Dimethenamid	73.2	0.15	0.04	2.7	3.69	-3
Dimethoate	69.2	1.1	0.30	4.3	6.21	19
Dimethylvinphos-(E)	70	0.12	0.03	3.8	5.43	-3
Dimethylvinphos-(Z)	70.7	0.12	0.03	3.7	5.23	-19
Diniconazole	70.2	0.36	0.10	2.9	4.13	8
Dinitramine	66.4	0.59	0.14	2.2	3.31	28
Dioxathion	73.6	4.9	1.37	2.9	3.94	15
Diphenamid	72.8	0.16	0.03	2.8	3.85	21
Diphenylamine	68.4	0.23	0.07	3.1	4.53	-9
Dithiopyr	73.9	0.19	0.04	2	2.71	-5
Edifenphos	74.6	0.13	0.03	3.6	4.83	-4
Endosulfan_alpha	69.5	0.51	0.15	2.1	3.02	22
Endosulfan_beta	67	0.45	0.10	5.3	7.91	21
Endosulfan_sulfate	66.2	0.41	0.10	6.6	9.97	36
Endrin	70.1	0.1	0.03	1.8	2.57	26
EPN	69.8	0.7	0.15	1.6	2.29	-2
Epoxiconazole	70.9	0.18	0.04	2.5	3.53	30
EPTC	52.6	0.36	0.09	1.8	3.42	-8
Esprocarb	71	0.1	0.02	3.1	4.37	-4
Etaconazole_1	76	0.3	0.08	5	6.58	20
Etaconazole_2	75.8	0.3	0.09	2.4	3.17	16
Ethalfluralin	72.7	0.1	0.03	1.9	2.61	30
Ethion	71.4	0.1	0.02	2.5	3.50	21
Ethofumesate	70.1	0.2	0.05	2.6	3.71	27
Ethoprophos	70.9	0.2	0.05	3.6	5.08	46
Ethychlozate	72	0.28	0.07	4.2	5.83	19
Etofenprox	79.5	7.1	2.13	2.8	3.52	9
Etoxazole	74.9	1.1	0.31	2.6	3.47	21
Etridiazole	60	0.52	0.12	2.3	3.83	18
Etrimfos	73.5	0.1	0.03	3.2	4.35	18
Fenamidone	70.6	0.1	0.02	0.7	0.99	16
Fenamiphos	68.6	0.4	0.10	5.1	7.43	4
Fenarimol	76	0.21	0.05	3.7	4.87	10
Fenazaquin	74.3	0.5	0.11	2.6	3.50	8
Fenbuconazole	72.5	1.46	0.42	2.5	3.45	7
Fenchlorphos	71.2	0.2	0.06	2.4	3.37	25
Fenclorim	71.1	0.1	0.03	2.4	3.38	13
Fenfuram	69.5	0.19	0.05	1.8	2.59	50

Fenitrothion	69.1	0.31	0.09	4.5	6.51	7
Fenobucarb	71.1	2.2	0.57	3.3	4.64	-18
Fenothiocarb	73.9	0.9	0.24	1	1.35	24
Fenoxanil	75.5	0.14	0.04	1.6	2.12	-4
Fenoxycarb	70.1	0.4	0.10	1.6	2.28	42
Fenpropathrin	77.5	0.1	0.03	2.7	3.48	30
Fenson	68.8	0.22	0.04	2.7	3.92	27
Fenthion	67.9	0.14	0.03	2.6	3.83	36
Fenvalerate-1	76.6	0.19	0.05	2.9	3.79	28
Fenvalerate-2	77	0.19	0.05	4.3	5.58	14
Fipronil	72.3	0.1	0.03	5.5	7.61	10
Flamprop-isopropyl	75.8	0.28	0.07	2.9	3.83	-33
Flonicamid	54.4	0.24	0.06	6.1	11.21	19
Fluazifop_butyl	73.2	0.25	0.07	2.9	3.96	7
Fluchloralin	65.6	0.09	0.02	3.9	5.95	-26
Flucythrinate_1	77.1	0.36	0.08	3.8	4.93	39
Flucythrinate_2	76.1	0.36	0.07	3.4	4.47	19
Fludioxonil	71.3	0.1	0.02	4.6	6.45	27
Flufenpyr_ethyl	75.4	0.32	0.07	3.6	4.77	16
Flumetralin	74.5	0.26	0.07	2.5	3.36	17
Flumiclorac_pentyl	78.9	0.32	0.10	3.9	4.94	2
Flumioxazine	71.6	0.23	0.06	2	2.79	7
Fluopyram	75	0.12	0.03	3.7	4.93	-15
Fluorodifen	62	0.26	0.06	2.2	3.55	18
Flurochloridone	68.9	0.26	0.07	3	4.35	50
Flusilazole	68	0.1	0.03	4.9	7.21	4
Flutamone	76.2	0.45	0.10	3.5	4.59	-1
Fluthiacet_methyl	83.5	0.032	0.01	1.3	1.56	-8
Flutianil	75.8	0.16	0.05	2.7	3.56	3
Flutolanil	74.4	0.1	0.02	3.7	4.97	15
Flutriafol	69.4	0.04	0.01	2.6	3.75	-18
Fluvalinate-1	78.6	0.73	0.18	4.8	6.11	25
Fluvalinate-2	79	0.73	0.20	3.8	4.81	35
Folpet	74.1	4.5	1.13	2.8	3.78	29
Fonofos	70.9	2.3	0.58	3.7	5.22	10
Formothion	62.3	0.1	0.02	2.8	4.49	-10
Fosthiazate-1	67.7	0.37	0.08	6.8	10.04	-14
Fosthiazate-2	69.6	0.37	0.10	6.5	9.34	23
Fthalide	71	0.1	0.02	2.7	3.80	25
Furathiocarb	75.5	0.1	0.03	3.8	5.03	-14
Halfenprox	76.1	0.9	0.18	4.4	5.78	-16
Heptachlor	67.8	0.5	0.10	3.1	4.57	10

Heptachlor-epoxide	69.9	0.15	0.03	3.1	4.43	9
Heptenophos	72.9	0.35	0.08	3.2	4.39	48
Hexachlorbenzene	67.9	0.1	0.02	1.6	2.36	-2
Hexaconazole	72	0.1	0.03	3.5	4.86	9
Imazalil	66.7	0.1	0.02	1.6	2.40	29
Indanofan	72.4	0.37	0.11	4.9	6.77	-14
Indoxacarb	80.4	3.9	0.98	2.9	3.61	11
Iprobenfos	70.5	0.19	0.05	3.3	4.68	27
Iprodione	77.5	0.1	0.02	3.7	4.77	27
Iprovalicarb-1	74.6	2.7	0.78	3.4	4.56	-19
Iprovalicarb-2	72.1	2.7	0.54	3.4	4.72	-10
Isazofos	72.6	0.42	0.12	2.2	3.03	-3
Isofenphos	72.8	0.13	0.04	3.5	4.81	25
Isofenphos-methyl	72	0.06	0.01	3.4	4.72	6
Isopropalin	69.8	0.18	0.04	3.2	4.58	-17
Isoprothiolane	73.2	0.12	0.02	2.5	3.42	8
Isotianil	76.3	0.78	0.20	1.8	2.36	11
Isoxadifen-ethyl	70.8	0.26	0.07	2.7	3.81	53
Isoxanthion	67	0.01	0.00	3.1	4.63	-12
Kresoxim-methyl	74.1	0.39	0.10	2.5	3.37	12
Lactofen	74.7	0.77	0.22	2.7	3.61	11
Leptophos	73.4	0.23	0.07	2.6	3.54	29
Malathion	72.5	0.6	0.15	4.2	5.79	-10
Mecarbam	69.9	1.55	0.34	2.3	3.29	18
Mefenacet	75.7	0.1	0.02	3.2	4.23	-5
Mefenpyr-diethyl	73.9	0.3	0.09	3.5	4.74	13
Mepronil	74.1	0.33	0.08	2.6	3.51	-17
Metazachlor	73.2	0.12	0.03	2.9	3.96	14
Metconazole	73.9	1	0.22	1.9	2.57	-8
Methidathion	71	0.14	0.03	3.3	4.65	11
Methoprotryne	71.2	0.25	0.05	2.9	4.07	-16
Methoxychlor	68.1	2	0.60	2.3	3.38	14
Methyltrithion	69.7	0.39	0.08	5.1	7.32	-15
Metolachlor	70.7	0.26	0.06	3.1	4.38	7
Metrafenone	75	0.3	0.07	4.4	5.87	-6
Metribuzin	68.4	0.2	0.05	2.4	3.51	-4
MGK-264_1	76.1	0.36	0.09	2.5	3.29	8
MGK-264_2	71.6	0.36	0.11	3	4.19	44
Mirex	70	0.1	0.02	1.2	1.71	14
Molinate	76.7	2.9	0.75	3.1	4.04	22
Monolinuron	68.2	0.3	0.06	2.9	4.25	17
Myclobutanil	71.9	0.14	0.04	2.6	3.62	9

Napropamide	72.6	0.6	0.18	3.7	5.10	-15
Nitrapyrin	59.7	0.41	0.09	2.2	3.69	36
Nitrothal-isopropyl	65.8	0.42	0.13	3.8	5.78	-13
Nonachlor_cis	46.4	0.16	0.03	4.8	10.34	7
Nonachlor_trans	47.9	0.16	0.04	4.7	9.81	8
Norflurazon	74.9	0.31	0.07	3.4	4.54	23
Nuarimol	75.9	0.31	0.09	1.9	2.50	28
Ofurace	73.6	0.1	0.02	1.4	1.90	50
Oxadiazon	75.1	0.3	0.06	3	3.99	21
Oxadixyl	61.5	0.19	0.04	6.9	11.22	10
Oxyflofen	72.5	0.33	0.07	2.6	3.59	29
Paclobutrazole	69.5	0.41	0.10	3.3	4.75	23
Parathion-ethyl	67.7	0.1	0.03	4.2	6.20	16
Parathion-methyl	70.1	0.35	0.09	3.7	5.28	-2
Pebulate	61.7	0.01	0.00	2	3.24	20
Penconazole	70.7	0.13	0.04	3	4.24	21
Pendimethalin	67.1	0.24	0.05	4.3	6.41	17
Penflufen	66.4	0.2	0.05	2.9	4.37	-14
Pentachlorobezonitrile	72	0.29	0.07	3.5	4.86	16
Penthiopyrad	76.7	0.22	0.06	2.8	3.65	5
Pentoxazon	74.8	0.34	0.09	2.6	3.48	12
Permethrin-1	80.9	0.65	0.14	3	3.71	-8
Permethrin-2	77.9	0.65	0.16	2.2	2.82	14
Perthane	67.8	0.15	0.04	2.3	3.39	-1
Phenothrin	81	0.003	0.00	4.8	5.93	47
Phenthoate	72.5	0.38	0.08	2.4	3.31	-18
Phorate	63.5	0.1	0.03	1.6	2.52	19
Phosalone	73.3	0.51	0.13	3.7	5.05	26
Phosmet(PMP)	71.9	0.91	0.21	3.9	5.42	22
Phosphamidone	58.8	1.9	0.48	11.6	19.73	29
Picolinafen	72.9	0.12	0.03	3.2	4.39	-11
Picoxystrobin	74.2	0.12	0.03	3.2	4.31	5
Piperonyl butoxide	76.2	0.26	0.06	2.4	3.15	-5
Piperophos	69.4	1.1	0.30	2.3	3.31	7
Pirimiphos-ethyl	69.1	0.18	0.05	1.6	2.32	-14
Pirimiphos-methyl	70.8	0.18	0.05	2.1	2.97	8
Pretilachlor	74.7	0.17	0.05	3.9	5.22	21
Primicarb	68.7	0.16	0.04	3.5	5.09	23
Probenazole	78.1	0.3	0.08	4.9	6.27	28
Prochloraz	72.6	1.1	0.30	3.5	4.82	42
Procymidone	75	1.2	0.25	2.7	3.60	-20
Profenofos	70.1	0.5	0.15	1.3	1.85	-20

Profluralin	71.1	0.46	0.12	3.7	5.20	-19
Prometon	68.3	0.66	0.15	2.6	3.81	-12
Prometryn	72.8	0.26	0.06	1.1	1.51	29
Pronamide	72.9	0.006	0.00	3.8	5.21	13
Propachlor	71.1	0.13	0.04	2.3	3.23	26
Propanil	73.7	0.15	0.04	2.1	2.85	5
Propazine	70.9	0.85	0.18	2.1	2.96	-11
Propetamphos	72.7	0.53	0.14	4	5.50	22
Propham	66.9	0.66	0.17	5.7	8.52	-48
Propiconazole-1	70.2	0.57	0.12	1.9	2.71	-13
Propiconazole-2	69.9	0.51	0.14	1.5	2.15	22
Propisochlor	74.1	0.9	0.25	3	4.05	-3
Prothiophos	72.8	0.21	0.06	2.5	3.43	-9
Pyracabolid	71.7	8.6	2.49	2	2.79	20
Pyraclofos	73.7	0.23	0.07	4.6	6.24	27
Pyrazophos	74.9	0.73	0.15	5.5	7.34	-14
Pyridaben	77.1	0.23	0.06	2.7	3.50	22
Pyridalyl	80.6	0.19	0.06	2.5	3.10	46
Pyridaphenthion	69.8	0.37	0.10	1.7	2.44	-10
Pyrifenox 1	60.8	0.86	0.24	1.6	2.63	29
Pyrifenox 2	65.1	5.33	1.44	5.1	7.83	26
Pyrimidifen	79.7	0.21	0.06	3.3	4.14	21
Pyriminobac-methyl(E)	74.3	0.1	0.02	1.9	2.56	13
Quinalphos	69.9	0.81	0.16	2.5	3.58	-18
Quinoxyfen	74.8	0.23	0.05	2	2.67	-17
Quintozene	66.5	0.36	0.10	4.9	7.37	33
Sectumeton	70.3	0.26	0.07	4.2	5.97	26
Silafluofen	79.7	0.12	0.03	2.4	3.01	13
Simeconazole	70.1	0.13	0.04	2.7	3.85	-10
Simetryn	69	1.6	0.37	3.7	5.36	9
Spiromesifen	77.6	0.16	0.04	3.3	4.25	46
Spiroxamine 1	68.8	1.12	0.24	3	4.36	-8
Spiroxamine_2	69.6	1.12	0.26	3.1	4.45	-14
Sulfotep	72.2	0.26	0.05	3.3	4.57	-20
Sulprofos	75.2	0.008	0.00	2.6	3.46	8
TCMTB	70.7	0.02	0.01	5.9	8.35	12
Tebuconazole	75.6	0.33	0.08	1.9	2.51	39
Tebufenpyrad	74.9	0.23	0.06	2.2	2.94	7
Tebupirimfos	70.6	0.19	0.04	4.3	6.09	14
Tefluthrin	75.9	0.13	0.04	3.1	4.08	16
Terbacil	69.4	0.9	0.22	3.5	5.04	2
Terbufos	67.3	0.4	0.11	1.7	2.53	9

Terbumeton	69.9	0.62	0.14	2.5	3.58	24
Terbuthylazine	73.7	0.26	0.08	1.9	2.58	-2
Terbutryn	74.3	0.32	0.08	2.9	3.90	45
Tetrachlorvinphos	72.1	0.15	0.03	3.2	4.44	-18
Tetraconazole	69.8	0.06	0.02	4.6	6.59	10
Tetradifon	75.7	0.17	0.04	2.2	2.91	25
Tetramethrin-1	81.6	0.86	0.21	3.7	4.53	3
Tetramethrin-2	75.2	0.86	0.25	2.8	3.72	-11
Tetrasul	73.2	0.08	0.02	1.7	2.32	-7
Thiazopyr	74.7	2	0.40	3.1	4.15	-13
Thifluzamide	73.9	0.26	0.06	2.5	3.38	27
Thiometon	49.2	0.23	0.05	9	18.29	-13
Thionazin	70	0.35	0.09	3.5	5.00	-12
Tolclofos_methyl	69.3	0.3	0.06	3.3	4.76	22
Tolfenpyrad	78	0.23	0.05	4.2	5.38	-3
Tolylfluanid	76.4	5.65	1.13	2.4	3.14	-9
Tralomethrin-1	65.3	1.82	0.38	5.4	8.27	12
Tralomethrin-2	77.3	1.82	0.38	5.2	6.73	29
Triadimefon	70.2	0.23	0.06	2.7	3.85	39
Triadimenol	70.7	1.6	0.37	3.4	4.81	-7
Triallate	70.7	0.26	0.08	3.7	5.23	-17
Triazophos	78.3	2.6	0.62	13.8	17.62	-11
Tribufos	71.9	0.005	0.00	2.6	3.62	24
Tridiphane	71.6	0.63	0.18	2.7	3.77	-14
Triflumizole	71.6	0.5	0.12	3.1	4.33	40
Triflumuron	75.4	0.2	0.05	2.5	3.32	12
Trifluralin	71	0.12	0.03	3.8	5.35	-11
Uniconazole	72.5	0.55	0.14	3.6	4.97	26
Vernlolate	60.2	0.01	0.00	2.1	3.49	-16
Vinclozoline	75.7	0.25	0.05	2.2	2.91	21
Zoxamide	74	0.07	0.02	3.2	4.32	4

Table S4. Average recovery(%), Limit of quantification(ng/g), SD(%), RSD(%) and matrix effect for the GC-MSMS method applied to maize.

Compound	maize					
	Average recovery	LOQ	LOD	SD	RSD	Matrix Effect
	(%)	(ng/g)	(ng/g)	(%)	(%)	(%)
2,6-Diisoporpylnaphthalene	65.3	4.09	1.23	2.0	3.06	-4
Acetochlor	76.6	0.09	0.03	3.0	3.92	-6
Acibenzola_s_methyl	73.8	4.1	0.90	1.7	2.30	30
Acrinathrin_1	73	0.63	0.16	5.1	6.99	-7
Acrinathrin_2	76.1	0.7	0.17	7.8	10.25	5
Alachlor	76	0.16	0.03	3.0	3.95	16
Aldrin	54.9	0.1	0.02	1.8	3.28	30
Allethrin-1	78	1.2	0.31	1.1	1.41	-9
Allethrin-2	72.8	1.2	0.29	4.1	5.63	6
Allidochlor	61.4	0.03	0.01	1.4	2.28	18
Ametryn	70.9	0.01	0.00	2.3	3.24	27
Anilofos	74.2	0.1	0.02	2.7	3.64	32
Aramit-1	66.5	0.19	0.05	3.4	5.11	7
Aramit-2	77.1	0.19	0.05	0.9	1.17	20
Aspon	73.2	0.09	0.02	2.5	3.42	-3
Atrazine	70.6	0.16	0.03	1.6	2.27	10
Azaconazole	68.3	0.19	0.05	3.4	4.98	16
Azinphos-ethyl	74.5	1.1	0.31	6.3	8.46	16
Azinphos-methyl	70.5	0.1	0.03	3.9	5.53	6
Benalaxyl	74.5	0.19	0.06	3	4.03	24
Benodanil	71.2	0.12	0.03	5	7.02	8
Benoxacor	73.9	0.45	0.09	2.2	2.98	-6
Benzoylprop_ethyl	75	0.35	0.09	1.7	2.27	8
BHC_alpha	66.6	0.17	0.04	2.5	3.75	22
BHC_beta	68.4	0.17	0.05	2.1	3.07	14
BHC_delta	68.8	0.17	0.03	2.7	3.92	26
BHC_gamma	66.6	0.17	0.04	2.5	3.75	25
Bifenox	70.1	0.21	0.05	4.6	6.56	12
Bifenthrin	68.4	0.13	0.03	1.7	2.49	5
Bromacil	77.1	0.1	0.03	2.5	3.24	26
Bromobutide	73.5	1.3	0.31	3.7	5.03	-5
Bromophos-ethyl	68.5	0.13	0.03	2.2	3.21	38
Bromophos-methyl	71.2	0.2	0.05	2.8	3.93	29
Bromopropylate	72.6	0.21	0.06	1.7	2.34	20

Bupirimate	75.9	0.16	0.04	3.8	5.01	20
Butachlor	74.2	0.6	0.16	5.5	7.41	3
Butafenacil	70.7	0.4	0.10	2.2	3.11	21
Butralin	74.5	0.19	0.04	4.4	5.91	24
Butylate	60.7	0.39	0.08	1.7	2.80	19
Cadusafos	73.2	0.15	0.03	2.7	3.69	38
Captan	72.9	4	0.92	1.4	1.92	4
Carbophenothion	67.3	0.1	0.02	3.5	5.20	6
Chinomethionat	65.5	0.21	0.06	3.2	4.89	6
Chlorbenside	59.6	0.17	0.05	4.4	7.38	-10
Chlorbufam	74.4	1.26	0.35	0.8	1.08	10
Chlordane_1	62.3	0.33	0.10	1.1	1.77	14
Chlordane_2	65.8	0.33	0.10	3	4.56	-7
Chlorethoxyfos	68.4	0.74	0.17	2.1	3.07	6
Chlorfenapyr	72.4	1.2	0.28	5	6.91	16
Chlorfenson	70.4	0.15	0.04	2.3	3.27	55
Chlorfluazuron	70	0.2	0.05	8.7	12.43	-6
Chlorflurenol_methyl	72.4	0.22	0.05	2.7	3.73	17
Chlornitrofen	67.6	0.23	0.06	7.3	10.80	27
Chlorobenzilate	69.6	0.13	0.03	4.1	5.89	-8
Chloroneb	66	1.65	0.35	1.1	1.67	6
Chloropropylate	70.1	0.15	0.05	3.9	5.56	13
Chloroxuron	67.1	0.3	0.08	3.5	5.22	-2
Chlorpropham	74.1	0.25	0.05	1.8	2.43	29
Chlorpyrifos	72.5	0.41	0.08	2.8	3.86	16
Chlorpyrifos-methyl	73.4	0.12	0.03	1	1.36	13
Chlorthal-dimethyl	75.4	0.06	0.02	1.5	1.99	29
Chlorthalonil	73.9	3	0.84	11.4	15.43	2
Chlorthion	71.5	0.25	0.07	4.2	5.87	3
Chlorthiophos_1	72.3	0.22	0.06	2.8	3.87	29
Chlorthiophos_2	71.6	0.22	0.05	2.1	2.93	-10
Chlozolinate	71	0.31	0.09	3.9	5.49	18
Cinidone_ethyl	78.3	0.31	0.08	5.5	7.02	17
Cinmethylin	77	1.75	0.40	3.4	4.42	17
Clomazon	72.7	0.08	0.02	1.7	2.34	3
Clomeporp	73.8	0.14	0.04	2.8	3.79	23
Coumaphos	72.8	3.5	0.98	2.8	3.85	-7
Crotoxyphos	72.8	0.15	0.04	4.1	5.63	11
Cyanazine	74.2	0.15	0.04	3	4.04	26
Cyanophos	73.8	0.08	0.02	2.6	3.52	9
Cycloate	68.2	0.02	0.00	2.5	3.67	11
Cyflufenamid	75.9	3	0.84	5	6.59	26

Cyfluthrin-1	69.1	0.9	0.23	1.3	1.88	29
Cyfluthrin-2	72.2	0.9	0.26	2	2.77	17
Cyfluthrin-3	72.9	0.9	0.21	2.9	3.98	15
Cyfluthrin-4	69.7	0.9	0.20	2.5	3.59	29
Cyhalofop-butyl	75.9	0.75	0.15	1.7	2.24	26
Cyhalothrin-1	71.6	0.9	0.22	2.9	4.05	7
Cyhalothrin-2	71.6	0.9	0.24	4	5.59	24
Cypermethrin-1	66.9	1	0.26	2.4	3.59	13
Cypermethrin-2	68.7	1	0.24	3.8	5.53	17
Cypermethrin-3	67.7	1	0.26	2.2	3.25	9
Cypermethrin-4	67.6	1	0.20	4.2	6.21	-2
Cyprazine	71.3	0.16	0.05	4.9	6.87	30
Cyproconazole-1	70.5	0.5	0.12	4.7	6.67	21
Cyproconazole-2	69.6	0.5	0.14	5.7	8.19	12
Cyprodinil	73.1	0.1	0.03	3.2	4.38	41
DDD_pp	65.5	0.1	0.03	2.1	3.21	14
DDE_pp	59.8	3.5	1.02	2.3	3.85	19
DDT_op	59.5	0.1	0.02	2.5	4.20	-3
DDT_pp	61.4	0.1	0.03	1.4	2.28	9
Deltamethrin-1	57.4	0.1	0.03	5.2	9.06	27
Deltamethrin-2	72.5	0.1	0.03	3.9	5.38	-9
Demeton_O	61.3	0.3	0.07	2	3.26	-9
Demeton_S	64.2	0.15	0.04	4.7	7.32	7
Demeton_S_methylsulfone	4.5	1.36	0.39	3.2	71.11	51
Desmetryn	72.5	7.6	1.82	3.9	5.38	15
Diafor	75	0.65	0.15	4.3	5.73	28
Diallate-1	71.6	0.11	0.03	2.4	3.35	16
Diallate-2	68.4	0.11	0.03	1.5	2.19	11
Diazinon	72.6	0.25	0.07	3.1	4.27	29
Dichlofenthion	70.6	0.15	0.03	1.7	2.41	6
Dichlofluanid	73.1	7.5	2.25	2.9	3.97	-16
Dichlormid	65	0.32	0.06	0.8	1.23	8
Dichlorvos	52.8	8.1	2.11	1	1.89	6
Diclobutrazole	71	0.35	0.09	5.5	7.75	15
Diclofop_methyl	77.2	0.23	0.06	1	1.30	27
Dicloran	72.5	0.41	0.09	3.6	4.97	11
Dicofol	67	0.16	0.03	2.2	3.28	34
Dieldrin	65.5	0.6	0.15	3.6	5.50	26
Diethatyl-ethyl	72.8	0.13	0.03	7	9.62	24
Diethofencarb	72	0.25	0.07	3.2	4.44	8
Difenoconazole-1	72.6	0.31	0.06	2	2.75	19
Difenoconazole-2	72.9	0.31	0.09	1.3	1.78	20

Diflufenican	75	0.23	0.07	1.6	2.13	12
Dimepiperate	73.2	3.1	0.68	4	5.46	4
Dimethachlor	73.8	0.55	0.13	2.8	3.79	5
Dimethametryn	72.4	0.13	0.03	2	2.76	14
Dimethenamid	74.9	0.12	0.03	2.1	2.80	8
Dimethoate	92.8	1.6	0.34	37.9	40.84	33
Dimethylvinphos-(E)	72.5	0.11	0.03	2.8	3.86	21
Dimethylvinphos-(Z)	74.4	0.11	0.03	4.3	5.78	22
Diniconazole	71.3	0.33	0.07	5.6	7.85	12
Dinitramine	72.3	0.52	0.16	2.4	3.32	17
Dioxathion	75.3	4.45	0.89	2.5	3.32	12
Diphenamid	73.6	0.17	0.05	1.6	2.17	14
Diphenylamine	69.8	0.22	0.05	2.1	3.01	12
Dithiopyr	77.2	0.17	0.05	1.9	2.46	-6
Edifenphos	74.4	0.13	0.04	3.7	4.97	5
Endosulfan_alpha	66.4	0.63	0.16	7.2	10.84	28
Endosulfan_beta	63.5	0.41	0.08	4.1	6.46	5
Endosulfan_sulfate	61.3	0.336	0.08	6.1	9.95	15
Endrin	62.1	0.1	0.03	4.5	7.25	36
EPN	74.2	0.5	0.10	3.9	5.26	26
Epoxiconazole	71.2	0.22	0.06	3.5	4.92	4
EPTC	56.8	0.4	0.10	1.6	2.82	6
Esprocarb	70.8	0.1	0.03	2.9	4.10	4
Etaconazole_1	75.7	0.35	0.08	3.9	5.15	21
Etaconazole_2	75.5	0.35	0.07	1.4	1.85	3
Ethalfluralin	76.9	0.1	0.03	3.4	4.42	45
Ethion	72.8	0.1	0.03	6	8.24	36
Ethofumesate	73.7	0.18	0.05	4.2	5.70	28
Ethoprophos	73.3	0.1	0.03	3	4.09	-10
Ethychlozate	68.3	0.33	0.08	4.2	6.15	36
Etofenprox	69.7	6.54	1.31	1.5	2.15	39
Etoxazole	76.5	1	0.27	2	2.61	21
Etridiazole	61.8	0.51	0.14	1.5	2.43	20
Etrimfos	75.6	0.1	0.03	1.7	2.25	21
Fenamidone	72.1	0.1	0.03	2.8	3.88	28
Fenamiphos	71.1	0.1	0.02	11.7	16.46	8
Fenarimol	72.4	0.2	0.04	5	6.91	-13
Fenazaquin	71.8	0.7	0.14	2.3	3.20	30
Fenbuconazole	70.3	1.1	0.32	2	2.84	14
Fenchlorphos	70.4	0.3	0.08	0.7	0.99	-10
Fenclorim	69	0.08	0.02	2.5	3.62	-6
Fenfuram	75	0.12	0.03	1.8	2.40	19

Fenitrothion	77.7	0.35	0.08	18.6	23.94	11
Fenobucarb	70.9	2.52	0.66	2.2	3.10	-17
Fenothiocarb	69.5	1	0.22	2.7	3.88	24
Fenoxanil	76.5	0.11	0.03	2.8	3.66	30
Fenoxycarb	71.8	0.4	0.11	12.4	17.27	26
Fenpropathrin	76.2	0.1	0.02	0.8	1.05	5
Fenson	68.7	0.16	0.03	1.2	1.75	-14
Fenthion	72.8	0.12	0.03	2.1	2.88	28
Fenvalerate-1	73.7	0.19	0.06	1.1	1.49	33
Fenvalerate-2	68.4	0.19	0.05	3.3	4.82	11
Fipronil	75.7	0.1	0.03	3.1	4.10	7
Flamprop-isopropyl	74.2	0.29	0.07	3.3	4.45	23
Flonicamid	34.4	0.26	0.05	12.1	35.17	-6
Fluazifop_butyl	73.7	0.3	0.09	2.6	3.53	36
Fluchloralin	74.3	0.08	0.02	5.7	7.67	29
Flucythrinate_1	71.3	0.37	0.09	2.4	3.37	30
Flucythrinate_2	74.8	0.37	0.11	1.3	1.74	-13
Fludioxonil	72.7	0.1	0.03	2.2	3.03	32
Flufenpyr_ethyl	72.5	0.35	0.07	5.6	7.72	8
Flumetralin	70.9	0.3	0.07	9.3	13.12	20
Flumiclorac_pentyl	78.3	0.35	0.07	2.1	2.68	15
Flumioxazine	75.9	0.26	0.08	3.4	4.48	33
Fluopyram	74.6	0.15	0.05	2.9	3.89	26
Fluorodifen	71.8	0.2	0.05	9.2	12.81	43
Flurochloridone	69.6	0.2	0.04	3.3	4.74	28
Flusilazole	73	0.1	0.03	4.4	6.03	36
Flutamone	73.9	0.52	0.10	3.4	4.60	55
Fluthiacet_methyl	83.9	0.025	0.01	6.7	7.99	25
Flutianil	74.7	0.14	0.03	1.2	1.61	18
Flutolanil	69.6	0.1	0.02	1.2	1.72	-8
Flutriafol	68	0.026	0.01	10.9	16.03	9
Fluvalinate-1	73.9	0.74	0.22	2.3	3.11	24
Fluvalinate-2	72.7	0.74	0.20	2.5	3.44	23
Folpet	74.1	4.2	1.26	2.6	3.51	34
Fonofos	72.5	2.6	0.60	1.9	2.62	-22
Formothion	69.3	0.1	0.03	7.8	11.26	30
Fosthiazate-1	70.3	0.36	0.09	5	7.11	50
Fosthiazate-2	71	0.36	0.08	3.5	4.93	20
Fthalide	70.2	0.1	0.03	2.3	3.28	5
Furathiocarb	71.4	0.1	0.02	2.7	3.78	-14
Halfenprox	60.6	0.6	0.15	5.7	9.41	-7
Heptachlor	61.9	0.5	0.12	1.4	2.26	28

Heptachlor-epoxide	65.8	0.15	0.03	1.9	2.89	-7
Heptenophos	72.6	0.32	0.09	2.3	3.17	-2
Hexachlorbenzene	57.3	0.1	0.03	2.8	4.89	-3
Hexaconazole	72.9	0.1	0.03	5.9	8.09	24
Imazalil	56.3	0.1	0.02	7.9	14.03	6
Indanofan	71.5	0.32	0.09	3.9	5.45	9
Indoxacarb	76.8	3.2	0.74	0.3	0.39	-4
Iprobenfos	75.4	0.13	0.04	3.1	4.11	-4
Iprodione	75.7	0.1	0.03	4.4	5.81	7
Iprovalicarb-1	71.2	2.8	0.76	5	7.02	-9
Iprovalicarb-2	72.4	2.8	0.56	6.3	8.70	12
Isazofos	74.4	0.35	0.09	2	2.69	24
Isofenphos	74.7	0.13	0.04	2	2.68	15
Isofenphos-methyl	73.1	0.07	0.02	3.6	4.92	14
Isopropalin	72.9	0.17	0.04	5	6.86	48
Isoprothiolane	73	0.11	0.03	2.2	3.01	5
Isotianil	72.4	0.65	0.18	2.1	2.90	23
Isoxadifen-ethyl	74.2	0.31	0.08	4.2	5.66	23
Isoxanthion	75.3	0.01	0.00	8.9	11.82	47
Kresoxim-methyl	73.3	0.37	0.10	2.7	3.68	-9
Lactofen	74.3	0.75	0.18	8.7	11.71	25
Leptophos	66.6	0.22	0.04	1.7	2.55	39
Malathion	74.8	0.5	0.11	4.7	6.28	3
Mecarbam	68.5	1.46	0.42	2.5	3.65	17
Mefenacet	73.8	0.1	0.03	2.7	3.66	3
Mefenpyr-diethyl	75.3	0.26	0.07	1.7	2.26	-8
Mepronil	73	0.31	0.09	4	5.48	51
Metazachlor	74.7	0.16	0.05	1.7	2.28	5
Metconazole	73.4	0.9	0.22	2.9	3.95	24
Methidathion	75.9	0.13	0.04	3.7	4.87	18
Methoprotryne	71.8	0.26	0.06	3.9	5.43	-13
Methoxychlor	68.7	1.6	0.32	1.6	2.33	-9
Methyltrithion	71.5	0.36	0.09	3.3	4.62	7
Metolachlor	73.1	0.23	0.06	2.1	2.87	-2
Metrafenone	73.8	0.27	0.07	0.5	0.68	21
Metribuzin	72.8	0.2	0.06	3.8	5.22	27
MGK-264_1	76.3	0.37	0.08	4.5	5.90	25
MGK-264_2	74.7	0.37	0.11	2	2.68	10
Mirex	48.7	0.1	0.02	3	6.16	22
Molinate	66.4	2.8	0.73	1.8	2.71	-10
Monolinuron	72.6	0.28	0.07	3.2	4.41	15
Myclobutanil	70.8	0.14	0.03	4.4	6.21	16

Napropamide	75.1	0.3	0.09	4.7	6.26	21
Nitrapyrin	60.7	0.51	0.12	1.1	1.81	-6
Nitrothal-isopropyl	74	0.39	0.09	6.8	9.19	5
Nonachlor_cis	59.7	0.16	0.03	3.2	5.36	15
Nonachlor_trans	58.1	0.16	0.04	2.8	4.82	16
Norflurazon	73.6	0.3	0.06	2.8	3.80	11
Nuarimol	71.4	0.32	0.07	2.7	3.78	15
Ofurace	73.7	0.1	0.02	4	5.43	-6
Oxadiazon	75.7	0.1	0.03	1.5	1.98	9
Oxadixyl	26.9	0.19	0.04	8.5	31.60	-4
Oxyflofen	73.7	0.36	0.10	11.4	15.47	3
Paclobutrazole	72.2	0.42	0.08	5.2	7.20	30
Parathion-ethyl	74.1	0.1	0.03	6	8.10	-10
Parathion-methyl	76.4	0.36	0.10	3.2	4.19	3
Pebulate	63.7	0.01	0.00	1.5	2.35	27
Penconazole	73.5	0.13	0.03	2.4	3.27	-5
Pendimethalin	76.4	0.2	0.05	5.4	7.07	28
Penflufen	76.3	0.23	0.05	3.1	4.06	8
Pentachlorobezonitrile	69.2	0.22	0.06	2.8	4.05	20
Penthiopyrad	75.7	0.21	0.06	4.5	5.94	-7
Pentoxazon	74.3	0.35	0.08	1.6	2.15	22
Permethrin-1	71	0.6	0.16	2	2.82	21
Permethrin-2	67.7	0.6	0.15	4.5	6.65	-16
Perthane	66.9	0.14	0.03	1.8	2.69	4
Phenothrin	66.9	0.029	0.01	3.4	5.08	21
Phenthoate	76.5	0.35	0.08	3.5	4.58	17
Phorate	70.1	0.1	0.03	3.1	4.42	-3
Phosalone	72.3	0.52	0.15	3.3	4.56	17
Phosmet(PMP)	73.8	0.88	0.20	3.9	5.28	8
Phosphamidone	49.6	1.8	0.38	11.8	23.79	8
Picolinafen	76.2	0.13	0.03	1.4	1.84	7
Picoxystrobin	73	0.16	0.04	5.3	7.26	9
Piperonyl butoxide	74.9	0.26	0.07	2	2.67	18
Piperophos	73.8	1	0.21	4.1	5.56	21
Pirimiphos-ethyl	74	0.18	0.04	2.3	3.11	-6
Pirimiphos-methyl	71.3	0.18	0.05	7	9.82	28
Pretilachlor	75.8	0.17	0.04	1.4	1.85	23
Primicarb	71.7	0.15	0.04	2.2	3.07	16
Probenazole	79.5	0.3	0.06	3.5	4.40	14
Prochloraz	68.6	1.1	0.29	2.2	3.21	29
Procymidone	75.4	1.2	0.32	2.7	3.58	27
Profenofos	75.9	0.4	0.09	2.8	3.69	13

Profluralin	74.9	0.39	0.09	3.3	4.41	-14
Prometon	70.4	0.64	0.15	3.9	5.54	8
Prometryn	72.9	0.23	0.06	3.1	4.25	15
Pronamide	73.5	0.006	0.00	2	2.72	10
Propachlor	70.1	0.16	0.04	2.2	3.14	-7
Propanil	76.1	0.13	0.04	4.3	5.65	-5
Propazine	74.2	0.82	0.20	2.6	3.50	2
Propetamphos	72.6	0.56	0.15	3.1	4.27	7
Propham	57.7	0.65	0.17	3.5	6.07	-6
Propiconazole-1	70	0.52	0.12	4.7	6.71	13
Propiconazole-2	73.6	0.52	0.14	1.5	2.04	19
Propisochlor	75.6	0.9	0.27	2.3	3.04	9
Prothiophos	66.1	0.22	0.05	3.7	5.60	26
Pyracabolid	72.3	7.9	1.74	3.4	4.70	26
Pyraclofos	75.1	0.25	0.06	6.2	8.26	27
Pyrazophos	75.5	0.71	0.19	6.5	8.61	14
Pyridaben	71.6	0.25	0.06	2.1	2.93	32
Pyridalyl	61.1	0.16	0.04	2.6	4.26	23
Pyridaphenthion	72.9	0.37	0.11	4.4	6.04	12
Pyrifenox 1	61.8	0.86	0.21	6	9.71	10
Pyrifenox 2	58.3	5.52	1.21	5.5	9.43	29
Pyrimidifen	74.7	0.26	0.08	1.3	1.74	5
Pyriminobac-methyl(E)	77.6	0.2	0.05	2.5	3.22	51
Quinalphos	69.3	0.8	0.20	3.3	4.76	31
Quinoxyfen	70.4	0.2	0.06	1.5	2.13	21
Quintozene	63.4	0.33	0.08	3.5	5.52	24
Sectumeton	74.2	0.26	0.05	4.1	5.53	28
Silafluofen	64.8	0.13	0.03	2.4	3.70	25
Simeconazole	71.3	0.11	0.03	3.1	4.35	9
Simetryn	70.6	2	0.56	4.5	6.37	5
Spiromesifen	75.4	0.16	0.04	1.3	1.72	30
Spiroxamine 1	68.8	1.11	0.24	3.5	5.09	5
Spiroxamine_2	68.8	1.11	0.32	5.2	7.56	5
Sulfotep	75.3	0.23	0.05	2.7	3.59	-10
Sulprofos	70.4	0.01	0.00	3.8	5.40	22
TCMTB	72	0.019	0.01	11.5	15.97	-6
Tebuconazole	71	0.4	0.10	1.8	2.54	17
Tebufenpyrad	75.9	0.25	0.06	1.5	1.98	4
Tebupirimfos	71.8	0.16	0.04	1.9	2.65	8
Tefluthrin	72.6	0.12	0.04	2.5	3.44	-21
Terbacil	70.9	0.87	0.21	2.4	3.39	31
Terbufos	70.2	0.4	0.09	3.2	4.56	24

Terbumeton	71.9	0.62	0.14	2.6	3.62	15
Terbuthylazine	69.8	0.25	0.06	4.5	6.45	-5
Terbutryn	73.3	0.36	0.08	3.7	5.05	6
Tetrachlorvinphos	70.6	0.12	0.02	3.5	4.96	14
Tetraconazole	73	0.05	0.01	5.4	7.40	12
Tetradifon	70.5	0.1	0.02	2.1	2.98	23
Tetramethrin-1	79.1	0.88	0.24	5.3	6.70	28
Tetramethrin-2	75.3	0.88	0.24	4	5.31	15
Tetrasul	55.5	0.06	0.02	3.2	5.77	15
Thiazopyr	73.2	1.2	0.29	4.4	6.01	22
Thifluzamide	70.8	0.3	0.08	4.8	6.78	30
Thiometon	68.1	0.25	0.05	3	4.41	-8
Thionazin	70.8	0.36	0.09	2.4	3.39	-3
Tolclofos_methyl	72	0.36	0.10	2.1	2.92	10
Tolfenpyrad	76.6	0.21	0.04	1.8	2.35	22
Tolylfluanid	71.8	5.9	1.59	3.4	4.74	4
Tralomethrin-1	55.1	1.43	0.40	8.8	15.97	22
Tralomethrin-2	73.4	1.43	0.40	3.9	5.31	5
Triadimefon	76.2	0.25	0.06	3.3	4.33	25
Triadimenol	72.6	2	0.58	1.9	2.62	22
Triallate	69.5	0.22	0.05	1.7	2.45	3
Triazophos	73.9	2.65	0.74	4.6	6.22	22
Tribufos	70.5	0.005	0.00	5.1	7.23	5
Tridiphane	69	0.64	0.15	2.4	3.48	-9
Triflumizole	72.7	0.52	0.14	1.7	2.34	-9
Triflumuron	72.6	0.5	0.11	0.6	0.83	-5
Trifluralin	76.4	0.12	0.03	4.3	5.63	-3
Uniconazole	72.1	0.56	0.12	4.8	6.66	-4
Vernlolate	63	0.01	0.00	1	1.59	5
Vinclozoline	72.7	0.23	0.07	0.7	0.96	6
Zoxamide	70.1	0.06	0.01	1	1.43	24

Table S5. Average recovery(%), Limit of quantification(ng/g), SD(%), RSD(%) and matrix effect for the GC-MSMS method applied to chilli pepper.

	chilli pepper					
Compound	Average recovery	LOQ	LOD	SD	RSD	Matrix Effect
	(%)	(ng/g)	(ng/g)	(%)	(%)	(%)
2,6-Diisoporpylnaphthalene	74.5	4.1	0.98	1.7	2.28	13
Acetochlor	79.9	0.1	0.03	1.6	2.00	19
Acibenzola_s_methyl	83.7	4.6	1.24	11.9	14.22	7
Acrinathrin_1	95.7	0.9	0.19	4.1	4.28	13
Acrinathrin_2	102.3	1.02	0.29	8.1	7.92	17
Alachlor	84.9	0.2	0.05	0.5	0.59	-16
Aldrin	76.7	0.1	0.02	4.0	5.22	25
Allethrin-1	65.6	1.3	0.26	10.8	16.46	25
Allethrin-2	75.3	1.3	0.29	9.5	12.62	18
Allidochlor	70.1	0.03	0.01	6.6	9.42	23
Ametryn	84.8	0.01	0.00	4.8	5.66	24
Anilofos	103.6	0.1	0.03	10.1	9.75	9
Aramit-1	84.2	0.19	0.05	5.2	6.18	17
Aramit-2	102.8	0.19	0.04	7.4	7.20	22
Aspon	87.7	0.09	0.02	6.9	7.87	28
Atrazine	70.9	0.15	0.04	7.0	9.87	-7
Azaconazole	79.3	0.2	0.05	2.9	3.66	25
Azinphos-ethyl	104.5	1.1	0.26	9.3	8.90	3
Azinphos-methyl	97.6	0.1	0.02	11.8	12.09	26
Benalaxyl	84.8	1.2	0.25	4.7	5.54	52
Benodanil	98.9	0.12	0.03	4.9	4.95	14
Benoxacor	84.5	0.32	0.10	3.4	4.02	-10
Benzoylprop_ethyl	85.1	0.3	0.08	4.7	5.52	28
BHC_alpha	79.3	0.17	0.05	4.6	5.80	46
BHC_beta	81.1	0.17	0.05	2	2.47	8
BHC_delta	80.6	0.17	0.04	5.5	6.82	30
BHC_gamma	75.7	0.17	0.04	1.6	2.11	25
Bifenox	104	0.21	0.05	8.7	8.37	-8
Bifenthrin	88	0.13	0.03	4.6	5.23	4
Bromacil	82.4	0.8	0.18	1.1	1.33	9
Bromobutide	81.1	1.3	0.34	4.4	5.43	5
Bromophos-ethyl	83.8	0.12	0.04	8.5	10.14	-9
Bromophos-methyl	84.6	0.2	0.04	1.2	1.42	10
Bromopropylate	89.9	0.21	0.06	6.7	7.45	16

Bupirimate	90.1	0.16	0.04	5.2	5.77	-2
Butachlor	90.2	0.9	0.26	1.9	2.11	30
Butafenacil	99.6	0.41	0.11	8	8.03	15
Butralin	79.2	0.24	0.06	2.6	3.28	29
Butylate	61.7	0.36	0.08	2	3.24	52
Cadusafos	82.8	0.16	0.04	3	3.62	-16
Captan	55.1	5.4	1.57	39	70.78	9
Carbophenothion	91.7	0.06	0.02	4.2	4.58	18
Chinomethionat	64.6	0.21	0.05	6.6	10.22	-9
Chlorbenside	97.2	0.18	0.04	7.3	7.51	30
Chlorbufam	86.7	1.25	0.34	2.2	2.54	18
Chlordane_1	79.3	0.31	0.08	5.3	6.68	-5
Chlordane_2	79.2	0.31	0.07	11.9	15.03	26
Chlorethoxyfos	79.2	0.75	0.15	2.2	2.78	25
Chlorfenapyr	86.6	1.2	0.24	1	1.15	10
Chlorfenson	75.9	0.12	0.02	10.7	14.10	21
Chlorfluazuron	63.5	0.2	0.04	7.9	12.44	-9
Chlorflurenol_methyl	81.8	0.21	0.06	1.4	1.71	28
Chlornitrofen	86.8	0.22	0.05	4.7	5.41	24
Chlorobenzilate	91.9	0.12	0.03	6.2	6.75	-16
Chloroneb	75.6	1.66	0.33	3	3.97	-13
Chloropropylate	88.3	0.13	0.03	4	4.53	10
Chloroxuron	84	0.31	0.08	3	3.57	29
Chlorpropham	78.1	0.25	0.07	6.6	8.45	12
Chlorpyrifos	83.7	0.41	0.12	2.1	2.51	39
Chlorpyrifos-methyl	82.5	0.12	0.03	1.2	1.45	-20
Chlorthal-dimethyl	81.9	0.07	0.02	4	4.88	8
Chlorthalonil	28	3.1	0.81	36.4	130.00	16
Chlorthion	86	0.21	0.04	6.3	7.33	4
Chlorthiophos_1	87.7	0.22	0.04	4.6	5.25	8
Chlorthiophos_2	86.6	0.22	0.05	3.8	4.39	29
Chlozolinate	79.6	0.3	0.07	7.4	9.30	-15
Cinidone_ethyl	93.8	0.31	0.08	8.5	9.06	8
Cinmethylin	91.5	1.69	0.49	2.4	2.62	29
Clomazon	77.8	0.08	0.02	2.3	2.96	-9
Clomeporp	92.3	0.14	0.04	5.6	6.07	23
Coumaphos	99.6	3.41	0.99	8	8.03	-8
Crotoxyphos	78.7	0.15	0.03	4.3	5.46	-14
Cyanazine	89.2	0.17	0.04	6.9	7.74	3
Cyanophos	91.3	0.08	0.02	2.9	3.18	48
Cycloate	76.7	0.03	0.01	2.7	3.52	-5
Cyflufenamid	88.2	2	0.50	1.1	1.25	24

Cyfluthrin-1	89.1	1	0.22	4.5	5.05	25
Cyfluthrin-2	91.4	1	0.25	3.8	4.16	39
Cyfluthrin-3	95.8	1	0.30	10	10.44	25
Cyfluthrin-4	96.2	1	0.21	2.2	2.29	17
Cyhalofop-butyl	88.2	1	0.30	4.4	4.99	11
Cyhalothrin-1	89.7	0.8	0.16	6.1	6.80	25
Cyhalothrin-2	92.7	0.8	0.22	9.7	10.46	15
Cypermethrin-1	92.7	1.1	0.22	6.3	6.80	22
Cypermethrin-2	98.2	1.1	0.31	6.7	6.82	10
Cypermethrin-3	91.5	1.1	0.26	7.2	7.87	6
Cypermethrin-4	99.5	1.1	0.25	9.6	9.65	5
Cyprazine	91.7	0.17	0.04	8	8.72	25
Cyproconazole-1	88	0.5	0.10	7.4	8.41	20
Cyproconazole-2	84.8	0.5	0.15	6.3	7.43	-8
Cyprodinil	88.5	0.1	0.02	4.5	5.08	16
DDD_pp	82.8	0.1	0.03	2.5	3.02	48
DDE_pp	87.5	4	1.20	5.9	6.74	2
DDT_op	74.4	0.1	0.03	4.5	6.05	35
DDT_pp	83	0.1	0.02	3.7	4.46	-6
Deltamethrin-1	88	0.1	0.03	30.7	34.89	-8
Deltamethrin-2	95.7	0.1	0.03	7.2	7.52	36
Demeton_O	64.7	0.31	0.09	7.8	12.06	24
Demeton_S	67	0.14	0.04	13.5	20.15	12
Demeton_S_methylsulfone	0.4	1.25	0.29	0.3	75.00	6
Desmetryn	82.2	7.6	1.82	0.4	0.49	15
Diafor	104.4	0.77	0.22	8.4	8.05	9
Diallate-1	81.4	0.11	0.03	4.1	5.04	14
Diallate-2	85	0.11	0.02	1.1	1.29	50
Diazinon	79.2	0.26	0.08	2.6	3.28	8
Dichlofenthion	84.5	0.14	0.03	4.7	5.56	19
Dichlofluanid	60.8	7.2	1.87	9.3	15.30	25
Dichlormid	63.6	0.36	0.09	3.4	5.35	30
Dichlorvos	49.2	7.9	2.13	2.1	4.27	-25
Diclobutrazole	67.9	0.38	0.11	14.5	21.35	6
Diclofop_methyl	85.9	0.19	0.06	4.1	4.77	11
Dicloran	88	0.39	0.08	0.5	0.57	14
Dicofol	91.7	0.16	0.04	2.5	2.73	11
Dieldrin	73.4	0.49	0.12	1.9	2.59	24
Diethatyl-ethyl	86	0.14	0.03	6.6	7.67	-10
Diethofencarb	91.2	0.3	0.08	4.2	4.61	6
Difenoconazole-1	115.7	0.33	0.09	20.1	17.37	29
Difenoconazole-2	113.4	0.33	0.09	20.1	17.72	-7

Diflufenican	87.5	0.21	0.06	2.4	2.74	25
Dimepiperate	86	2.56	0.72	7.3	8.49	16
Dimethachlor	85.1	0.7	0.21	3	3.53	26
Dimethametryn	82	0.15	0.04	4.3	5.24	15
Dimethenamid	81.9	0.11	0.03	2.9	3.54	8
Dimethoate	92	0.8	0.18	13.8	15.00	23
Dimethylvinphos-(E)	89.8	0.12	0.03	1.5	1.67	25
Dimethylvinphos-(Z)	84.3	0.12	0.03	5.7	6.76	15
Diniconazole	96.5	0.39	0.11	13.5	13.99	29
Dinitramine	83.7	0.55	0.15	9.2	10.99	25
Dioxathion	93.2	4.8	1.34	5.5	5.90	30
Diphenamid	85.4	0.13	0.03	5.6	6.56	50
Diphenylamine	66.4	0.21	0.06	1.4	2.11	-16
Dithiopyr	85.6	0.12	0.03	2.8	3.27	23
Edifenphos	87.3	0.11	0.03	3.1	3.55	9
Endosulfan_alpha	95.4	0.42	0.11	2.9	3.04	-9
Endosulfan_beta	76.9	0.51	0.11	5.1	6.63	21
Endosulfan_sulfate	86.1	0.32	0.08	12.3	14.29	19
Endrin	84.4	0.1	0.03	3.2	3.79	5
EPN	97.9	0.5	0.15	8.4	8.58	15
Epoxiconazole	99.1	0.16	0.04	7.1	7.16	14
EPTC	56.8	0.31	0.09	2.5	4.40	6
Esprocarb	81.7	0.1	0.03	3.1	3.79	5
Etaconazole_1	97.1	0.26	0.06	10	10.30	-19
Etaconazole_2	86.1	0.26	0.07	3.7	4.30	28
Ethalfluralin	89.1	0.1	0.02	3.4	3.82	13
Ethion	82.7	0.1	0.02	4	4.84	23
Ethofumesate	94.3	0.25	0.06	3.6	3.82	27
Ethoprophos	85.3	0.2	0.05	5.2	6.10	4
Ethychlozate	77.6	0.31	0.09	7.1	9.15	25
Etofenprox	85.1	7.3	2.19	4.9	5.76	19
Etoxazole	94.5	1.1	0.23	6.6	6.98	28
Etridiazole	64.9	0.46	0.11	0.9	1.39	28
Etrimfos	87.9	0.1	0.03	2.6	2.96	21
Fenamidone	100.6	0.1	0.03	6.2	6.16	15
Fenamiphos	83.7	0.1	0.03	3.7	4.42	14
Fenarimol	89.2	0.18	0.04	5.8	6.50	26
Fenazaquin	83.4	0.5	0.14	4.1	4.92	-6
Fenbuconazole	77.7	1.3	0.34	5.9	7.59	-10
Fenchlorphos	81.8	0.25	0.08	4.9	5.99	14
Fenclorim	78.8	0.05	0.01	2.8	3.55	26
Fenfuram	5.6	0.12	0.03	2.3	41.07	46

Fenitrothion	87.2	0.35	0.09	2.7	3.10	2
Fenobucarb	81.5	2.7	0.81	3.2	3.93	50
Fenothiocarb	80.8	0.9	0.25	6.6	8.17	9
Fenoxanil	84.9	0.11	0.03	9.2	10.84	24
Fenoxycarb	53.4	0.7	0.20	2.7	5.06	28
Fenpropathrin	112.3	0.1	0.02	5.3	4.72	27
Fenson	80.6	0.3	0.09	3.7	4.59	-13
Fenthion	83.5	0.13	0.04	6.1	7.31	24
Fenvalerate-1	93.4	0.2	0.06	6.6	7.07	-19
Fenvalerate-2	99.4	0.2	0.04	8.3	8.35	14
Fipronil	98.3	0.1	0.02	8.7	8.85	24
Flamprop-isopropyl	82.1	0.23	0.06	3.9	4.75	17
Flonicamid	13.5	0.26	0.05	7.2	53.33	15
Fluazifop_butyl	68.3	0.21	0.05	14.8	21.67	-12
Fluchloralin	89.7	0.06	0.02	2.8	3.12	55
Flucythrinate_1	102.7	0.35	0.09	7.1	6.91	19
Flucythrinate_2	101.5	0.35	0.09	7.2	7.09	13
Fludioxonil	113.7	0.1	0.03	7	6.16	23
Flufenpyr_ethyl	92.5	0.4	0.08	9.3	10.05	12
Flumetralin	73.3	0.21	0.05	10.1	13.78	22
Flumiclorac_pentyl	100.4	0.39	0.08	10.4	10.36	22
Flumioxazine	113.4	0.3	0.06	7.1	6.26	25
Fluopyram	89.1	0.1	0.03	8.7	9.76	39
Fluorodifen	80.3	0.16	0.04	2.6	3.24	5
Flurochloridone	95.1	0.23	0.07	4.2	4.42	26
Flusilazole	118.9	0.1	0.03	5.1	4.29	29
Flutamone	97.4	0.5	0.11	7	7.19	15
Fluthiacet_methyl	101	0.03	0.01	10.1	10.00	36
Flutianil	95.2	0.2	0.04	5	5.25	-2
Flutolanil	11.7	0.1	0.03	1.7	14.53	-10
Flutriafol	79.9	0.03	0.01	4	5.01	28
Fluvalinate-1	104.8	0.74	0.17	10.5	10.02	13
Fluvalinate-2	104	0.74	0.17	11	10.58	29
Folpet	66.5	4.9	1.13	1.1	1.65	47
Fonofos	83.8	2.6	0.70	4.5	5.37	-2
Formothion	78.6	0.1	0.03	9.5	12.09	1
Fosthiazate-1	94.5	0.35	0.08	5.3	5.61	3
Fosthiazate-2	90.6	0.35	0.08	7.3	8.06	19
Fthalide	78.6	0.1	0.02	2.2	2.80	22
Furathiocarb	97.1	0.1	0.02	10.3	10.61	-3
Halfenprox	90.8	1	0.25	4.6	5.07	20
Heptachlor	78.2	0.7	0.19	2.8	3.58	43

Heptachlor-epoxide	78.2	0.15	0.04	11.6	14.83	38
Heptenophos	77.7	0.33	0.09	2.8	3.60	23
Hexachlorbenzene	71.4	0.1	0.02	5.7	7.98	7
Hexaconazole	78.4	0.1	0.03	4.4	5.61	-16
Imazalil	83.2	0.1	0.02	3.3	3.97	-15
Indanofan	83.3	0.37	0.10	5.4	6.48	-10
Indoxacarb	77.9	3	0.81	3	3.85	12
Iprobenfos	94.7	0.13	0.03	8.4	8.87	26
Iprodione	84	0.1	0.03	4.5	5.36	21
Iprovalicarb-1	82.5	2.4	0.62	5.4	6.55	30
Iprovalicarb-2	83.9	2.4	0.72	4.8	5.72	8
Isazofos	86.8	0.45	0.09	4.2	4.84	24
Isofenphos	86.6	0.11	0.02	4.9	5.66	16
Isofenphos-methyl	91.1	0.03	0.01	6.6	7.24	7
Isopropalin	83	0.17	0.04	5.5	6.63	17
Isoprothiolane	83.4	0.15	0.04	4.3	5.16	25
Isotianil	89.3	0.63	0.18	3.9	4.37	13
Isoxadifen-ethyl	87.4	0.27	0.07	8.1	9.27	6
Isoxanthion	85.3	0.01	0.00	2.3	2.70	21
Kresoxim-methyl	91.4	0.35	0.09	7.6	8.32	23
Lactofen	115.5	0.74	0.16	15.8	13.68	9
Leptophos	86.3	0.16	0.04	5.6	6.49	19
Malathion	85.6	0.4	0.10	2.3	2.69	8
Mecarbam	89.2	1.56	0.39	4.5	5.04	8
Mefenacet	91.6	0.1	0.02	4.6	5.02	7
Mefenpyr-diethyl	90.6	0.31	0.09	5.5	6.07	20
Mepronil	81	0.31	0.07	5.3	6.54	16
Metazachlor	83.3	0.1	0.03	6.9	8.28	16
Metconazole	95	0.6	0.16	9	9.47	8
Methidathion	90.7	0.12	0.03	3.8	4.19	14
Methoprotryne	69.8	0.23	0.05	5.7	8.17	18
Methoxychlor	88.1	1.5	0.39	6.1	6.92	6
Methyltrithion	79	0.35	0.09	4.2	5.32	14
Metolachlor	82.2	0.23	0.05	3.6	4.38	-6
Metrafenone	87.1	0.26	0.08	7.2	8.27	49
Metribuzin	86.9	0.21	0.05	5.2	5.98	19
MGK-264_1	86.5	0.4	0.10	5.4	6.24	-4
MGK-264_2	80.7	0.4	0.11	4.4	5.45	-22
Mirex	77.7	0.1	0.03	4.4	5.66	8
Molinate	69.9	2.5	0.75	2.3	3.29	16
Monolinuron	83.8	0.23	0.06	4.4	5.25	15
Myclobutanil	97.8	0.14	0.03	9.4	9.61	30

Napropamide	85.3	0.4	0.10	2.2	2.58	9
Nitrapyrin	65.4	0.43	0.09	1.5	2.29	17
Nitrothal-isopropyl	89.3	0.41	0.09	6.4	7.17	21
Nonachlor_cis	77.3	0.18	0.05	4.8	6.21	24
Nonachlor_trans	81.8	0.18	0.04	3.8	4.65	15
Norflurazon	83.6	0.26	0.07	3.2	3.83	20
Nuarimol	91.1	0.33	0.10	10.1	11.09	10
Ofurace	87.3	0.3	0.07	5.4	6.19	35
Oxadiazon	96.6	0.1	0.03	6.7	6.94	26
Oxadixyl	80.1	0.16	0.04	3.3	4.12	15
Oxyflofen	67.9	0.35	0.08	14.5	21.35	-4
Paclobutrazole	93.8	0.37	0.10	8.2	8.74	19
Parathion-ethyl	89	0.1	0.02	6.2	6.97	-8
Parathion-methyl	87.2	0.38	0.08	2	2.29	-7
Pebulate	66.2	0.008	0.00	2.2	3.32	28
Penconazole	83.3	0.14	0.03	1.2	1.44	7
Pendimethalin	90.3	0.26	0.07	7.9	8.75	-9
Penflufen	88.3	0.21	0.05	3.9	4.42	15
Pentachlorobezonitrile	76.2	0.23	0.06	5.2	6.82	17
Penthiopyrad	1.6	0.25	0.07	0.6	37.50	7
Pentoxazon	87.1	0.36	0.10	5.6	6.43	20
Permethrin-1	83.7	0.55	0.15	4.5	5.38	3
Permethrin-2	86.8	0.55	0.15	5.8	6.68	2
Perthane	82.3	0.18	0.04	4.5	5.47	22
Phenothrin	96.1	0.031	0.01	13.3	13.84	-6
Phenthoate	83.6	0.35	0.10	4.6	5.50	5
Phorate	79.3	0.1	0.03	5.7	7.19	0
Phosalone	105.9	0.55	0.15	12.1	11.43	4
Phosmet(PMP)	98.8	0.86	0.24	9.6	9.72	24
Phosphamidone	43.4	1.6	0.37	29.2	67.28	-1
Picolinafen	91.7	0.18	0.05	5.2	5.67	20
Picoxystrobin	63.1	0.14	0.04	3	4.75	-5
Piperonyl butoxide	102.5	0.23	0.05	8.4	8.20	11
Piperophos	119.1	1.2	0.34	15.3	12.85	-7
Pirimiphos-ethyl	88.2	0.13	0.04	7.6	8.62	2
Pirimiphos-methyl	80.2	0.13	0.04	11.1	13.84	1
Pretilachlor	82.8	0.12	0.03	3.5	4.23	17
Primicarb	80.7	0.18	0.04	1.8	2.23	1
Probenazole	71.2	0.6	0.17	11.8	16.57	-1
Prochloraz	70.7	1.1	0.23	9.8	13.86	-4
Procymidone	87.7	1.1	0.24	4.4	5.02	30
Profenofos	77.3	0.4	0.09	2.6	3.36	-6

Profluralin	88.7	0.36	0.08	7.3	8.23	24
Prometon	82.3	0.65	0.18	3	3.65	29
Prometryn	74.5	0.29	0.06	13.6	18.26	14
Pronamide	79.9	0.006	0.00	3.2	4.01	-4
Propachlor	78.2	0.15	0.04	3.5	4.48	24
Propanil	82.5	0.15	0.03	6.7	8.12	20
Propazine	79.9	0.83	0.20	4.9	6.13	36
Propetamphos	93.9	0.54	0.15	6	6.39	20
Propham	75.3	0.62	0.16	12.9	17.13	41
Propiconazole-1	92.7	0.5	0.10	4.5	4.85	18
Propiconazole-2	80.4	0.5	0.14	9	11.19	10
Propisochlor	79.6	1	0.24	1.8	2.26	-8
Prothiophos	84.6	0.26	0.06	4	4.73	17
Pyracabolid	86.2	9.1	2.09	6	6.96	15
Pyraclofos	98.3	0.23	0.05	7.5	7.63	3
Pyrazophos	110.8	0.74	0.16	10	9.03	4
Pyridaben	87.2	0.21	0.04	4.5	5.16	23
Pyridalyl	97.5	0.16	0.04	4.9	5.03	26
Pyridaphenthion	120.3	0.39	0.10	14.9	12.39	-9
Pyrifenox 1	62.5	0.82	0.23	5.1	8.16	29
Pyrifenox 2	50.3	5.29	1.27	4.5	8.95	22
Pyrimidifen	98.8	0.23	0.06	6.2	6.28	19
Pyriminobac-methyl(E)	101.2	0.2	0.05	6.6	6.52	-15
Quinalphos	86.7	0.82	0.25	7.3	8.42	8
Quinoxyfen	80.5	0.26	0.08	2.6	3.23	21
Quintozene	78.4	0.39	0.08	4	5.10	15
Sectumeton	81.2	0.23	0.05	6.3	7.76	22
Silafluofen	87.2	0.17	0.03	2.8	3.21	9
Simeconazole	88.9	0.13	0.03	7.5	8.44	20
Simetryn	83.1	2.1	0.46	3.8	4.57	-4
Spiromesifen	79.8	0.2	0.05	7.5	9.40	-21
Spiroxamine 1	81.1	1.15	0.29	8.7	10.73	25
Spiroxamine_2	81.5	1.15	0.30	7.3	8.96	26
Sulfotep	90.4	0.21	0.04	4.1	4.54	30
Sulprofos	84.1	0.01	0.00	4.1	4.88	27
TCMTB	84.1	0.02	0.00	7.1	8.44	11
Tebuconazole	88.1	0.36	0.08	6.8	7.72	0
Tebufenpyrad	86.1	0.2	0.06	5.3	6.16	4
Tebupirimfos	85.5	0.12	0.03	5.2	6.08	28
Tefluthrin	81.3	0.11	0.03	3.2	3.94	1
Terbacil	89	0.84	0.24	5.9	6.63	-1
Terbufos	87.2	0.2	0.06	4.6	5.28	26

Terbumeton	82.4	0.65	0.16	6.6	8.01	-8
Terbuthylazine	82.8	0.21	0.06	5.6	6.76	24
Terbutryn	89.8	0.32	0.08	2.9	3.23	-8
Tetrachlorvinphos	86.7	0.13	0.03	2.1	2.42	-7
Tetraconazole	89.5	0.08	0.02	4.1	4.58	8
Tetradifon	80.1	0.14	0.03	4.7	5.87	24
Tetramethrin-1	101.1	0.87	0.19	14.6	14.44	4
Tetramethrin-2	92.8	0.87	0.22	5.4	5.82	27
Tetrasul	76	0.07	0.02	5.4	7.11	5
Thiazopyr	86.8	1.1	0.25	5.8	6.68	1
Thifluzamide	82.8	0.4	0.10	5.8	7.00	20
Thiometon	60.7	0.23	0.05	4.9	8.07	21
Thionazin	82.5	0.3	0.08	4.7	5.70	-1
Tolclofos_methyl	79.4	0.29	0.08	2.6	3.27	20
Tolfenpyrad	95.4	0.26	0.08	7.4	7.76	18
Tolylfluanid	71.3	5.23	1.52	5.5	7.71	19
Tralomethrin-1	140.2	1.8	0.52	42.3	30.17	2
Tralomethrin-2	91.4	1.8	0.38	3.2	3.50	-3
Triadimefon	78.9	0.24	0.07	4.3	5.45	-10
Triadimenol	97.8	1.4	0.29	8.5	8.69	-9
Triallate	80	0.24	0.06	4.3	5.38	14
Triazophos	98.2	2.55	0.61	4.4	4.48	10
Tribufos	86.3	0.005	0.00	9	10.43	24
Tridiphane	85.4	0.7	0.16	4.6	5.39	17
Triflumizole	90.4	0.53	0.14	6.9	7.63	-8
Triflumuron	84.8	0.3	0.09	4.7	5.54	6
Trifluralin	79.1	0.11	0.03	4.1	5.18	7
Uniconazole	81.4	0.52	0.14	3.7	4.55	12
Vernlolate	64.7	0.01	0.00	2	3.09	12
Vinclozoline	82.7	0.24	0.05	7.8	9.43	30
Zoxamide	110.5	0.07	0.02	32.5	29.41	19

Table S6. Correlation coefficient(R^2) of GC-MS/MS of 322 pesticides.

Compound	coffee	potato	maize	chilli pepper
2,6-Diisoporpylnaphthalene	0.9979	0.9968	0.9971	0.9995
Acetochlor	0.9852	0.9997	0.9969	0.9998
Acibenzola_s_methyl	0.9990	0.9898	0.9996	0.9964
Acrinathrin_1	0.9968	0.9993	0.9972	0.9984
Acrinathrin_2	0.9974	0.9980	0.9877	0.9992
Alachlor	0.9975	0.9994	0.9878	0.9992
Aldrin	0.9928	0.9993	0.9906	0.9985
Allethrin-1	0.9995	0.9991	0.9968	0.9997
Allethrin-2	0.9997	0.9994	0.9941	0.9982
Allidochlor	0.9989	0.9993	0.9979	0.9998
Ametryn	0.9906	0.9965	0.9951	0.9995
Anilofos	0.9975	0.9982	0.9849	0.9999
Aramit-1	0.9963	0.9993	0.9996	0.9999
Aramit-2	0.9847	0.9991	0.9966	0.9997
Aspon	0.9922	0.9990	0.9846	0.9995
Atrazine	0.9989	0.9999	0.9985	0.9997
Azaconazole	0.9989	0.9996	0.9968	0.9997
Azinphos-ethyl	0.9848	0.9998	0.9980	0.9997
Azinphos-methyl	0.9866	0.9991	0.9996	0.9996
Benalaxyl	0.9994	0.9994	0.9995	0.9989
Benodanil	0.9961	0.9999	0.9989	0.9990
Benoxacor	0.9980	0.9952	0.9970	0.9994
Benzoylprop_ethyl	0.9994	0.9999	0.9965	0.9994
BHC_alpha	0.9951	0.9959	0.9978	0.9986
BHC_beta	0.9884	0.9985	0.9944	0.9997
BHC_delta	0.9856	0.9993	0.9991	0.9999
BHC_gamma	0.9986	1.0000	0.9813	0.9976
Bifenox	0.9998	0.9996	0.9990	0.9998
Bifenthrin	0.9922	0.9999	0.9859	1.0000
Bromacil	0.9991	0.9992	0.9968	0.9999
Bromobutide	0.9920	0.9988	0.9941	0.9998
Bromophos-ethyl	0.9994	0.9993	0.9994	0.9999
Bromophos-methyl	0.9976	0.9997	0.9967	1.0000
Bromopropylate	0.9963	0.9993	0.9879	0.9998
Bupirimate	0.9975	0.9993	0.9994	0.9997
Butachlor	0.9970	0.9956	0.9974	0.9986
Butafenacil	0.9983	0.9990	0.9980	0.9993
Butralin	0.9978	0.9996	0.9992	0.9998
Butylate	0.9994	0.9994	0.9956	0.9997
Cadusafos	0.9995	0.9994	0.9928	0.9992
Captan	0.9989	0.9996	0.9904	0.9991
Carbophenothion	0.9928	0.9989	0.9996	0.9998
Chinomethionat	0.9996	0.9995	0.9993	0.9997
Chlorbenside	0.9998	0.9995	0.9979	0.9999
Chlorbufam	0.9927	0.9997	0.9941	1.0000
Chlordane_1	0.9954	0.9945	0.9841	0.9982
Chlordane_2	0.9965	0.9992	0.9976	0.9986
Chlorethoxyfos	0.9964	0.9988	0.9807	0.9985
Chlorfenapyr	0.9989	0.9994	0.9987	0.9997
Chlorfenson	0.9959	0.9984	0.9959	0.9999
Chlorfluazuron	0.9964	0.9989	0.9990	0.9996
Chlorflurenol_methyl	0.9956	0.9983	0.9989	0.9991
Chlornitrofen	0.9972	0.9989	0.9993	0.9996
Chlorobenzilate	0.9974	0.9819	0.9984	0.9899
Chloroneb	0.9987	0.9989	0.9993	0.9998
Chloropropylate	0.9984	0.9996	0.9987	0.9999
Chloroxuron	0.9981	0.9997	0.9971	0.9996

Chlorpropham	0.9984	0.9991	0.9953	0.9997
Chlorpyrifos	0.9959	0.9988	0.9929	0.9993
Chlorpyrifos-methyl	0.9972	0.9990	0.9881	0.9998
Chlorthal-dimethyl	0.9979	0.9987	0.9976	0.9999
Chlorthalonil	0.9978	0.9992	0.9900	0.9997
Chlorthion	0.9928	0.9985	0.9985	0.9998
Chlorthiophos_1	0.9906	0.9983	0.9976	0.9978
Chlorthiophos_2	0.9952	0.9996	0.9933	0.9998
Chlozolinate	0.9878	0.9995	0.9834	0.9999
Cinidone_ethyl	0.9992	0.9994	0.9989	0.9997
Cinmethylin	0.9961	0.9994	0.9979	0.9998
Clomazon	0.9944	0.9985	0.9908	0.9998
Clomeporp	0.9992	0.9996	0.9958	0.9996
Coumaphos	0.9958	0.9998	0.9972	0.9998
Crotoxyphos	0.9927	0.9994	0.9924	0.9997
Cyanazine	0.9934	0.9992	0.9992	0.9997
Cyanophos	0.9973	0.9995	0.9943	0.9999
Cycloate	0.9997	0.9992	0.9975	0.9995
Cyflufenamid	0.9983	0.9897	0.9977	0.9856
Cyfluthrin-1	0.9967	0.9956	0.9984	0.9986
Cyfluthrin-2	0.9988	0.9994	0.9971	0.9993
Cyfluthrin-3	0.9932	0.9995	0.9929	0.9995
Cyfluthrin-4	0.9997	0.9995	0.9992	1.0000
Cyhalofop-butyl	0.9985	0.9983	0.9993	0.9994
Cyhalothrin-1	0.9984	0.9996	0.9938	0.9996
Cyhalothrin-2	0.9996	0.9982	0.9977	0.9982
Cypermethrin-1	0.9936	0.9994	0.9963	0.9997
Cypermethrin-2	0.9937	0.9976	0.9911	0.9984
Cypermethrin-3	0.9993	0.9976	0.9991	0.9987
Cypermethrin-4	0.9975	0.9987	0.9930	0.9997
Cyprazine	0.9993	0.9995	0.9976	0.9998
Cyproconazole-1	0.9983	0.9992	0.9982	0.9999
Cyproconazole-2	0.9994	0.9926	0.9988	0.9919
Cyprodinil	0.9997	0.9989	0.9948	0.9983
DDD_pp	0.9930	0.9990	0.9991	0.9999
DDE_pp	0.9958	0.9994	0.9925	1.0000
DDT_op	0.9891	0.9995	0.9989	0.9998
DDT_pp	0.9986	0.9995	0.9974	0.9998
Deltamethrin-1	0.9992	0.9996	0.9993	0.9998
Deltamethrin-2	0.9949	0.9996	0.9989	1.0000
Demeton_O	0.9919	0.9992	0.9875	0.9998
Demeton_S	0.9949	0.9995	0.9964	0.9997
Demeton_S_methylsulfone	0.9992	0.9997	0.9975	1.0000
Desmetryn	0.9983	0.9997	0.9953	0.9998
Diafor	0.9976	0.9998	0.9991	0.9992
Diallate-1	0.9940	0.9991	0.9952	0.9990
Diallate-2	0.9996	0.9989	0.9991	0.9999
Diazinon	0.9953	0.9999	0.9970	0.9997
Dichlofenthion	0.9882	0.9986	0.9921	0.9998
Dichlofluanid	0.9959	0.9992	0.9984	0.9997
Dichlormid	0.9883	0.9972	0.9985	0.9986
Dichlorvos	0.9988	0.9997	0.9912	0.9996
Diclobutrazole	0.9994	0.9954	0.9996	0.9996
Diclofop_methyl	0.9964	0.9994	0.9953	0.9997
Dicloran	0.9978	0.9992	0.9975	0.9997
Dicofol	0.9976	0.9990	0.9893	0.9997
Dieldrin	0.9984	0.9991	0.9966	0.9999
Diethatyl-ethyl	0.9977	0.9990	0.9970	0.9994
Diethofencarb	0.9979	0.9998	0.9963	0.9993

Difenoconazole-1	0.9861	0.9995	0.9987	0.9999
Difenoconazole-2	0.9970	0.9982	0.9994	0.9986
Diflufenican	0.9958	0.9983	0.9958	0.9997
Dimepiperate	0.9992	0.9997	0.9965	0.9998
Dimethachlor	0.9988	0.9993	0.9952	0.9999
Dimethametryn	0.9992	0.9992	0.9965	0.9995
Dimethenamid	0.9984	0.9987	0.9961	0.9993
Dimethoate	0.9861	0.9994	0.9963	0.9999
Dimethylvinphos-(E)	0.9914	0.9993	0.9980	1.0000
Dimethylvinphos-(Z)	0.9979	0.9877	0.9979	0.9942
Diniconazole	0.9947	0.9994	0.9979	0.9999
Dinitramine	0.9820	0.9994	0.9966	0.9999
Dioxathion	0.9999	0.9996	0.9973	0.9998
Diphenamid	0.9966	0.9997	0.9981	0.9995
Diphenylamine	0.9998	0.9977	0.9905	0.9994
Dithiopyr	0.9906	0.9994	0.9919	0.9993
Edifenphos	0.9985	0.9999	0.9949	0.9999
Endosulfan_alphs	0.9999	0.9985	0.9931	0.9998
Endosulfan_beta	0.9997	0.9994	0.9975	0.9999
Endosulfan_sulfate	0.9928	0.9997	0.9845	0.9995
Endrin	0.9962	0.9995	0.9967	0.9999
EPN	0.9979	0.9993	0.9980	0.9998
Epoxiconazole	0.9953	0.9994	0.9924	0.9968
EPTC	0.9897	0.9989	0.9969	0.9997
Esprocarb	0.9999	0.9996	0.9978	0.9999
Etaconazole_1	0.9988	0.9997	0.9955	0.9977
Etaconazole_2	0.9989	0.9999	0.9939	0.9999
Ethalfluralin	0.9987	0.9991	0.9942	0.9998
Ethion	0.9997	0.9987	0.9989	0.9996
Ethofumesate	0.9988	0.9986	0.9965	0.9997
Ethoprophos	0.9993	0.9987	0.9982	0.9998
Ethychlozate	0.9960	0.9990	0.9879	0.9999
Etofenprox	0.9996	0.9998	0.9937	0.9979
Etoxazole	0.9923	0.9999	0.9841	0.9983
Etridiazole	0.9944	0.9979	0.9958	0.9997
Etrimfos	0.9993	0.9992	0.9938	1.0000
Fenamidone	0.9857	0.9997	0.9993	0.9995
Fenamiphos	0.9926	0.9997	0.9908	0.9996
Fenarimol	0.9996	0.9994	0.9987	0.9997
Fenazaquin	0.9992	0.9965	0.9957	0.9974
Fenbuconazole	0.9997	0.9997	0.9987	0.9995
Fenchlorphos	0.9989	0.9998	0.9913	0.9978
Fenclorim	0.9990	0.9990	0.9966	0.9997
Fenfuram	0.9973	0.9994	0.9863	0.9999
Fenitrothion	0.9992	0.9999	0.9952	0.9980
Fenobucarb	0.9959	0.9994	0.9972	0.9978
Fenothiocarb	0.9932	0.9998	0.9988	0.9999
Fenoxanil	0.9939	0.9992	0.9870	0.9995
Fenoxycarb	0.9973	0.9956	0.9986	0.9958
Fenpropathrin	0.9967	0.9998	0.9992	0.9997
Fenson	0.9974	0.9995	0.9983	0.9989
Fenthion	0.9991	0.9981	0.9964	0.9997
Fenvalerate-1	0.9996	0.9977	0.9981	0.9990
Fenvalerate-2	0.9943	0.9925	0.9988	0.9987
Fipronil	0.9970	0.9976	0.9926	0.9999
Flamprop-isopropyl	0.9990	0.9992	0.9969	0.9998
Flonicamid	0.9993	0.9995	0.9965	0.9999
Fluazifop_butyl	0.9955	0.9997	0.9987	0.9997
Fluchloralin	0.9997	0.9993	0.9990	0.9943

Flucythrinate_1	0.9975	0.9995	0.9993	0.9997
Flucythrinate_2	0.9938	0.9990	0.9907	0.9998
Fludioxonil	0.9991	0.9991	0.9910	0.9999
Flufenpyr_ethyl	0.9978	0.9980	0.9993	0.9946
Flumetralin	0.9980	0.9997	0.9993	0.9999
Flumiclorac_pentyl	0.9934	0.9987	0.9988	0.9997
Flumioxazine	0.9983	0.9998	0.9954	0.9999
Fluopyram	0.9947	0.9995	0.9964	0.9995
Fluorodifen	0.9990	0.9990	0.9951	0.9999
Flurochloridone	0.9991	0.9986	0.9884	0.9995
Flusilazole	0.9987	0.9982	0.9958	0.9974
Flutamone	0.9932	0.9989	0.9964	0.9999
Fluthiacet_methyl	0.9951	0.9995	0.9983	0.9999
Flutianil	0.9960	0.9991	0.9934	0.9998
Flutolanil	0.9975	0.9987	0.9911	0.9997
Flutriafol	0.9995	0.9995	0.9996	0.9996
Fluvalinate-1	0.9992	0.9990	0.9857	0.9998
Fluvalinate-2	0.9992	0.9984	0.9975	0.9998
Folpet	0.9918	0.9874	0.9971	0.9902
Fonofos	0.9886	0.9997	0.9901	0.9999
Formothion	0.9967	0.9991	0.9930	0.9997
Fosthiazate-1	0.9995	0.9997	0.9989	0.9997
Fosthiazate-2	0.9819	0.9997	0.9986	0.9997
Fthalide	0.9993	0.9990	0.9994	0.9987
Furathiocarb	0.9994	0.9998	0.9982	0.9996
Halfenprox	0.9988	0.9992	0.9976	0.9998
Heptachlor	0.9995	0.9936	0.9992	0.9905
Heptachlor-epoxide	0.9958	0.9993	0.9983	0.9997
Heptenophos	0.9956	0.9992	0.9978	0.9999
Hexachlorbenzene	0.9978	0.9994	0.9861	0.9999
Hexaconazole	0.9974	0.9991	0.9919	0.9996
Imazalil	0.9971	0.9985	0.9969	0.9988
Indanofan	0.9980	0.9991	0.9930	0.9998
Indoxacarb	0.9972	0.9987	0.9956	0.9999
Iprobenfos	0.9973	1.0000	0.9936	1.0000
Iprodione	0.9828	0.9991	0.9946	0.9999
Iprovalicarb-1	0.9996	0.9998	0.9995	1.0000
Iprovalicarb-2	0.9995	0.9998	0.9987	0.9999
Isazofos	0.9972	0.9994	0.9959	0.9997
Isofenphos	0.9958	0.9994	0.9974	0.9996
Isofenphos-methyl	0.9949	0.9988	0.9959	0.9997
Isopropalin	0.9984	0.9998	0.9973	0.9990
Isoprothiolane	0.9899	0.9990	0.9933	0.9999
Isotianil	0.9974	0.9991	0.9937	0.9998
Isoxadifen-ethyl	0.9986	0.9953	0.9984	0.9988
Isoxanthion	0.9973	0.9991	0.9944	0.9982
Kresoxim-methyl	0.9983	0.9988	0.9913	0.9998
Lactofen	0.9991	0.9999	0.9849	1.0000
Leptophos	0.9868	0.9993	0.9928	0.9997
Malathion	0.9997	0.9968	0.9982	0.9974
Mecarbam	0.9872	0.9956	0.9970	0.9965
Mefenacet	0.9996	0.9975	0.9929	0.9985
Mefenpyr-diethyl	0.9970	0.9988	0.9966	1.0000
Mepronil	0.9960	0.9986	0.9989	0.9996
Metazachlor	0.9964	0.9997	0.9995	1.0000
Metconazole	0.9873	0.9993	0.9932	0.9997
Methidathion	0.9987	0.9993	0.9984	0.9999
Methoprotryne	0.9977	0.9998	0.9908	1.0000
Methoxychlor	0.9974	0.9991	0.9967	0.9996

Methyltrithion	0.9907	0.9990	0.9952	0.9998
Metolachlor	0.9992	0.9998	0.9937	0.9993
Metrafenone	0.9996	0.9993	0.9993	0.9977
Metribuzin	0.9982	0.9999	0.9899	1.0000
MGK-264_1	0.9971	0.9987	0.9810	0.9996
MGK-264_2	0.9937	0.9998	0.9912	1.0000
Mirex	0.9968	0.9999	0.9915	0.9999
Molinate	0.9969	0.9992	0.9924	0.9995
Monolinuron	0.9996	0.9995	0.9958	0.9997
Myclobutanil	0.9915	0.9985	0.9978	0.9990
Napropamide	0.9844	0.9996	0.9970	0.9990
Nitrapyrin	0.9986	0.9993	0.9941	0.9996
Nitrothal-isopropyl	0.9981	0.9996	0.9998	0.9997
Nonachlor_cis	0.9999	0.9980	0.9951	0.9973
Nonachlor_trans	0.9989	0.9992	0.9944	0.9997
Norflurazon	0.9950	0.9992	0.9961	0.9996
Nuarimol	0.9997	0.9996	0.9995	0.9999
Ofurace	0.9989	0.9993	0.9982	0.9996
Oxadiazon	0.9994	0.9994	0.9987	0.9999
Oxadixyl	0.9981	0.9992	0.9960	0.9998
Oxyflofen	0.9989	0.9956	0.9971	0.9993
Paclobutrazole	0.9986	0.9991	0.9941	0.9998
Parathion-ethyl	0.9982	0.9992	0.9962	0.9999
Parathion-methyl	0.9995	0.9997	0.9984	0.9998
Pebulate	0.9991	0.9994	0.9921	0.9999
Penconazole	0.9991	0.9996	0.9976	0.9999
Pendimethalin	0.9951	0.9994	0.9920	0.9995
Penflufen	0.9975	0.9993	0.9975	0.9999
Pentachlorobezonitrile	0.9993	0.9990	0.9992	0.9996
Penthiopyrad	0.9985	0.9993	0.9987	0.9998
Pentoxazon	0.9934	0.9991	0.9997	0.9996
Permethrin-1	0.9947	0.9995	0.9949	0.9996
Permethrin-2	0.9962	0.9995	0.9982	0.9999
Perthane	0.9991	0.9996	0.9882	0.9999
Phenothrin	0.9994	0.9995	0.9989	0.9996
Phenthoate	0.9965	0.9996	0.9926	0.9996
Phorate	0.9982	0.9968	0.9972	0.9995
Phosalone	0.9997	0.9986	0.9989	0.9991
Phosmet(PMP)	0.9993	0.9991	0.9978	0.9995
Phosphamidone	0.9966	0.9993	0.9949	0.9992
Picolinafen	0.9998	0.9992	0.9931	0.9998
Picoxystrobin	0.9906	0.9993	0.9975	0.9999
Piperonyl butoxide	0.9985	0.9995	0.9845	0.9997
Piperophos	0.9999	0.9985	0.9967	0.9989
Pirimiphos-ethyl	0.9997	0.9992	0.9980	0.9997
Pirimiphos-methyl	0.9928	0.9996	0.9924	0.9999
Pretilachlor	0.9962	0.9994	0.9969	0.9998
Primicarb	0.9979	0.9994	0.9978	0.9997
Probenazole	0.9953	0.9962	0.9955	0.9985
Prochloraz	0.9897	0.9989	0.9939	0.9998
Procymidone	0.9999	0.9996	0.9942	0.9998
Profenofos	0.9988	0.9993	0.9989	1.0000
Profluralin	0.9989	0.9936	0.9965	0.9989
Prometon	0.9987	0.9989	0.9982	0.9992
Prometryn	0.9997	0.9990	0.9879	0.9984
Pronamide	0.9988	0.9976	0.9937	0.9991
Propachlor	0.9993	0.9997	0.9841	0.9994
Propanil	0.9960	0.9995	0.9958	0.9999
Propazine	0.9996	0.9992	0.9938	0.9999

Propetamphos	0.9923	0.9988	0.9993	0.9972
Propham	0.9944	0.9997	0.9908	0.9997
Propiconazole-1	0.9993	0.9983	0.9987	0.9999
Propiconazole-2	0.9857	0.9966	0.9957	0.9953
Propisochlor	0.9926	0.9989	0.9987	0.9999
Prothiophos	0.9996	0.9989	0.9913	0.9998
Pyracabolid	0.9992	0.9998	0.9966	0.9998
Pyraclofos	0.9997	0.9987	0.9863	0.9994
Pyrazophos	0.9989	0.9985	0.9952	0.9968
Pyridaben	0.9990	0.9956	0.9972	0.9995
Pyridalyl	0.9973	0.9998	0.9988	0.9998
Pyridaphenthion	0.9992	0.9995	0.9870	0.9964
Pyrifenox 1	0.9959	0.9981	0.9986	0.9984
Pyrifenox 2	0.9932	0.9977	0.9992	0.9992
Pyrimidifen	0.9939	0.9925	0.9983	0.9992
Pyriminobac-methyl(E)	0.9973	0.9976	0.9964	0.9985
Quinalphos	0.9967	0.9992	0.9981	0.9997
Quinoxyfen	0.9974	0.9995	0.9988	0.9982
Quintozene	0.9991	0.9997	0.9926	0.9998
Sectumeton	0.9920	0.9993	0.9969	0.9995
Silafluofen	0.9994	0.9995	0.9965	0.9999
Simeconazole	0.9976	0.9990	0.9987	0.9999
Simetryn	0.9963	0.9991	0.9990	0.9997
Spiromesifen	0.9975	0.9980	0.9993	0.9995
Spiroxamine 1	0.9970	0.9997	0.9907	0.9997
Spiroxamine_2	0.9983	0.9987	0.9949	0.9997
Sulfotep	0.9978	0.9998	0.9931	0.9997
Sulprofos	0.9994	0.9995	0.9975	0.9996
TCMTB	0.9995	0.9990	0.9845	0.9989
Tebuconazole	0.9989	0.9986	0.9967	0.9990
Tebufenpyrad	0.9928	0.9982	0.9980	0.9994
Tebupirimfos	0.9996	0.9989	0.9924	0.9994
Tefluthrin	0.9998	0.9995	0.9969	0.9986
Terbacil	0.9927	0.9991	0.9978	0.9997
Terbufos	0.9954	0.9987	0.9955	0.9999
Terbumeton	0.9965	0.9995	0.9939	0.9976
Terbuthylazine	0.9964	0.9990	0.9942	0.9998
Terbutryn	0.9989	0.9984	0.9989	1.0000
Tetrachlorvinphos	0.9959	0.9874	0.9965	0.9999
Tetraconazole	0.9964	0.9997	0.9982	0.9998
Tetradifon	0.9956	0.9991	0.9879	0.9999
Tetramethrin-1	0.9972	0.9997	0.9937	1.0000
Tetramethrin-2	0.9949	0.9997	0.9841	0.9998
Tetrasul	0.9919	0.9990	0.9958	0.9997
Thiazopyr	0.9949	0.9998	0.9938	0.9986
Thifluzamide	0.9992	0.9992	0.9993	0.9993
Thiometon	0.9983	0.9936	0.9908	0.9998
Thionazin	0.9976	0.9993	0.9987	0.9997
Tolclofos_methyl	0.9940	0.9992	0.9957	0.9992
Tolfenpyrad	0.9996	0.9994	0.9987	0.9991
Tolylfluanid	0.9953	0.9991	0.9913	0.9998
Tralomethrin-1	0.9882	0.9985	0.9966	0.9997
Tralomethrin-2	0.9959	0.9991	0.9863	0.9999
Triadimefon	0.9883	0.9987	0.9952	1.0000
Triadimenol	0.9988	1.0000	0.9972	0.9982
Triallate	0.9994	0.9991	0.9988	0.9986
Triazophos	0.9964	0.9998	0.9870	0.9985
Tribufos	0.9978	0.9998	0.9986	0.9997
Tridiphane	0.9976	0.9994	0.9992	0.9999

Triflumizole	0.9984	0.9994	0.9983	0.9996
Triflumuron	0.9973	0.9988	0.9964	0.9991
Trifluralin	0.9992	0.9998	0.9981	0.9996
Uniconazole	0.9959	0.9990	0.9988	0.9899
Vernlolate	0.9932	0.9991	0.9926	0.9998
Vinclozoline	0.9939	0.9953	0.9969	0.9999
Zoxamide	0.9973	0.9991	0.9965	0.9996

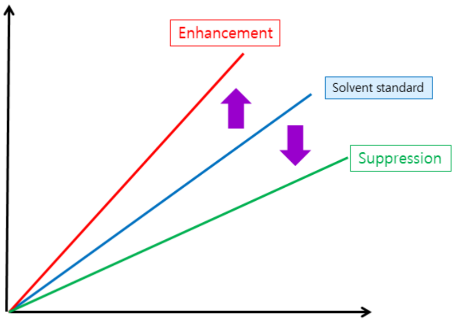

Figure S1. Enhancement and Suppression of slop in standard solutions by Matrix Effect.

Figure S2. Chromatogram analysis of pesticides of agricultural products.

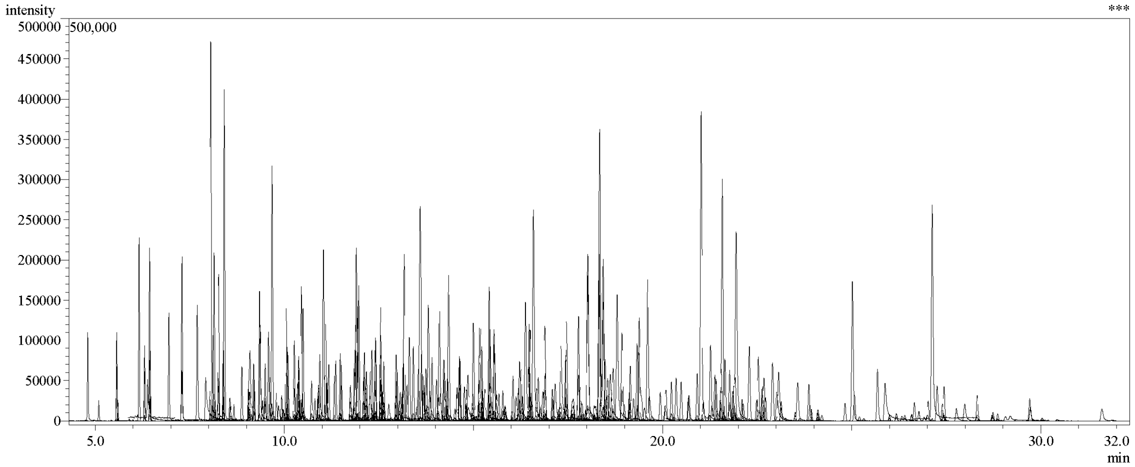

Quantitative Result Table

ID#	Name	R.Time	m/z	Area	Height	Conc.	Conc.Unit
1	Dichlorvos	4.804	109.00 > 79.00	106368	42736	100.000	ng/mL
2	Allidochlor	5.094	138.00 > 96.00	15085	6450	100.000	ng/mL
3	EPTC	5.568	128.00 > 86.00	96627	43208	100.000	ng/mL
4	Dichlormid	5.598	172.00 > 108.00	21967	9700	100.000	ng/mL
5	Butylate	6.160	156.00 > 57.00	168596	72934	100.000	ng/mL
6	Vernolate	6.305	128.00 > 86.00	97907	42280	100.000	ng /mL
7	Propham	6.384	119.00 > 91.00	40734	14587	100.000	ng/mL
8	Triflumuron	6.433	139.00 > 111.00	39469	4392	100.000	ng/mL
9	Pebulate	6.437	128.00 > 57.00	235819	97182	100.000	ng/mL
10	Nitrapyrin	6.448	194.00 > 133.00	70185	29177	100.000	ng/mL
11	Etridiazole	6.450	211.00 > 140.00	77977	33322	100.000	ng/mL
12	Chloroneb	6.947	206.00 > 191.00	156535	60400	100.000	ng/mL
13	Molinate	7.293	126.00 > 55.00	302991	119458	100.000	ng/mL
14	Heptenophos	7.699	124.00 > 89.00	209592	76188	100.000	ng/mL
15	Flonicamid	7.923	174.00 > 146.00	229767	37257	100.000	ng/mL
16	Thionazin	8.047	143.00 > 79.00	49487	19124	100.000	ng/mL
17	Fenobucarb	8.053	121.00 > 77.00	458256	175485	100.000	ng/mL
18	Propachlor	8.144	120.00 > 77.00	231649	87449	100.000	ng/mL
19	Demeton_O	8.112	171.00 > 115.00	29654	12049	100.000	ng/mL
20	Diphenylamine	8.256	169.00 > 66.00	77885	18689	100.000	ng/mL
21	Chlorethoxyfos	8.260	153.00 > 97.00	144390	61001	100.000	ng/mL
22	Ethoprophos	8.386	200.00 > 158.00	57046	21995	100.000	ng/mL
23	Cycloate	8.411	83.00 > 55.00	501563	187609	100.000	ng/mL
24	Chlorpropham	8.562	213.00 > 171.00	31021	9142	100.000	ng/mL
25	Ethalfluralin	8.667	276.00 > 202.00	21963	8942	100.000	ng/mL
26	Trifluralin	8.876	306.00 > 264.00	71269	27416	100.000	ng/mL
27	Sulfotep	9.052	238.00 > 146.00	31915	12384	100.000	ng/mL
28	Cadusafos	9.089	159.00 > 131.00	128474	34735	100.000	ng/mL
29	Diallate-1	9.187	234.00 > 150.00	61481	22943	100.000	ng/mL
30	Phorate	9.205	260.00 > 75.00	15468	5935	100.000	ng/mL
31	Fenclorim	9.347	224.00 > 189.00	224632	67206	100.000	ng/mL

ID#	Name	R.Time	m/z	Area	Height	Conc.	Conc.Unit
32	BHC_alpha	9.364	181.00 > 145.00	153074	55242	100.000	ng/mL
33	Diallate-2	9.422	234.00 > 150.00	22527	8461	100.000	ng/mL
34	Thiometon	9.492	125.00 > 47.00	35642	12412	100.000	ng/mL
35	Hexachlorbenzene	9.582	284.00 > 249.00	100689	36092	100.000	ng/mL
36	Demeton_S	9.621	170.00 > 114.00	4194	1595	100.000	ng/mL
37	Dicloran	9.670	206.00 > 176.00	39727	8437	100.000	ng/mL
38	2,6-Diisoporpylnaphthal	9.676	197.00 > 155.00	354764	128722	100.000	ng/mL
39	Dimethoate	9.693	143.00 > 111.00	4886	1433	100.000	ng/mL
40	Prometon	9.791	210.00 > 168.00	43151	14925	100.000	ng/mL
41	Chlorbufam	9.850	153.00 > 125.00	28753	7693	100.000	ng/mL
42	Atrazine	9.929	215.00 > 58.00	29160	9268	100.000	ng/mL
43	Monolinuron	9.964	214.00 > 61.00	18620	5579	100.000	ng/mL
44	Clomazon	10.049	204.00 > 107.00	103843	35404	100.000	ng/mL
45	Propazine	10.051	214.00 > 172.00	84546	29970	100.000	ng/mL
46	BHC_beta	10.076	181.00 > 145.00	125273	42688	100.000	ng/mL
47	Terbumeton	10.096	169.00 > 154.00	116116	36556	100.000	ng/mL
48	Probenazole	10.243	159.00 > 130.00	7681	2408	100.000	ng/mL
49	Dioxathion	10.261	125.00 > 97.00	51163	18082	100.000	ng/mL
50	BHC_gamma	10.263	181.00 > 145.00	137443	47695	100.000	ng/mL
51	Terbuthylazine	10.349	214.00 > 71.00	41732	14611	100.000	ng/mL
52	Propetamphos	10.364	138.00 > 110.00	80661	28906	100.000	ng/mL
53	Cyanophos	10.379	109.00 > 79.00	58499	19340	100.000	ng/mL
54	Terbufos	10.385	231.00 > 129.00	60262	21317	100.000	ng/mL
55	Quintozene	10.408	295.00 > 237.00	27739	10153	100.000	ng/mL
56	Profluralin	10.424	318.00 > 199.00	11975	4752	100.000	ng/mL
57	Pronamide	10.449	173.00 > 145.00	247329	76349	100.000	ng/mL
58	Pentachlorobezonitrile	10.463	275.00 > 240.00	34558	10947	100.000	ng/mL
59	Fonofos	10.494	137.00 > 109.00	199910	71046	100.000	ng/mL
60	Diazinon	10.720	137.00 > 84.00	50912	18332	100.000	ng/mL
61	Fluchloralin	10.807	306.00 > 264.00	33120	12302	100.000	ng/mL
62	Terbacil	10.885	161.00 > 144.00	44743	13396	100.000	ng/mL
63	Sectumeton	10.900	169.00 > 154.00	48539	14198	100.000	ng/mL
64	Fenfuram	10.917	201.00 > 109.00	118562	20219	100.000	ng/mL
65	BHC_delta	10.938	181.00 > 145.00	121233	38741	100.000	ng/mL
66	Dinitramine	10.929	261.00 > 195.00	6050	2445	100.000	ng/mL
67	Tefluthrin	11.035	177.00 > 127.00	297624	106517	100.000	ng/mL
68	Chlorthalonil	11.090	266.00 > 231.00	144071	44986	100.000	ng/mL
69	Isazofos	11.117	161.00 > 119.00	81003	28889	100.000	ng/mL
70	Triallate	11.128	268.00 > 226.00	38882	13893	100.000	ng/mL
71	Etrimfos	11.173	181.00 > 153.00	98324	34262	100.000	ng/mL
72	Tebupirimfos	11.325	261.00 > 137.00	38606	13802	100.000	ng/mL
73	Iprobenfos	11.358	204.00 > 91.00	69230	24590	100.000	ng/mL
74	Benoxacor	11.480	120.00 > 93.00	70725	23554	100.000	ng/mL
75	Formothion	11.483	224.00 > 196.00	1767	678	100.000	ng/mL
76	Primicarb	11.506	238.00 > 166.00	89498	29176	100.000	ng/mL
77	Desmetryn	11.746	213.00 > 58.00	44373	12867	100.000	ng/mL

ID#	Name	R.Time	m/z	Area	Height	Conc.	Conc.Unit
78	Propanil	11.835	217.00 > 161.00	37528	6111	100.000	ng/mL
79	Cyprazine	11.852	227.00 > 212.00	31597	11055	100.000	ng/mL
80	Dichlofenthion	11.875	279.00 > 223.00	138760	50054	100.000	ng/mL
81	Phosphamidone	11.880	127.00 > 109.00	3897	1405	100.000	ng/mL
82	Dimethachlor	11.902	134.00 > 105.00	258256	87142	100.000	ng/mL
83	Metribuzin	11.924	198.00 > 82.00	35488	11854	100.000	ng/mL
84	Dimethenamid	11.941	230.00 > 154.00	185982	64598	100.000	ng/mL
85	Bromobutide	11.968	119.00 > 91.00	334362	111301	100.000	ng/mL
86	Acetochlor	12.101	223.00 > 147.00	32459	11019	100.000	ng/mL
87	Spiroxamine 2	12.115	100.00 > 72.00	94630	31061	100.000	ng/mL
88	Vinclozoline	12.155	198.00 > 145.00	43824	15194	100.000	ng/mL
89	Parathion-methyl	12.165	263.00 > 109.00	41115	13428	100.000	ng/mL
90	Chlorpyrifos-methyl	12.171	286.00 > 93.00	44968	15019	100.000	ng/mL
91	Simetryn	12.261	213.00 > 198.00	5558	1941	100.000	ng/mL
92	Simeconazole	12.272	121.00 > 101.00	93911	27870	100.000	ng/mL
93	Tolclofos_methyl	12.315	265.00 > 250.00	120031	39787	100.000	ng/mL
94	Acibenzola_s_methyl	12.351	182.00 > 152.00	9043	2593	100.000	ng/mL
95	Heptachlor	12.403	100.00 > 65.00	105362	37221	100.000	ng/mL
96	Alachlor	12.411	188.00 > 160.00	94542	33995	100.000	ng/mL
97	Ametryn	12.417	227.00 > 170.00	35677	11152	100.000	ng/mL
98	Prometryn	12.538	241.00 > 226.00	15017	5095	100.000	ng/mL
99	Propisochlor	12.541	162.00 > 120.00	102887	35239	100.000	ng/mL
100	Cinmethylin	12.547	105.00 > 77.00	156506	54072	100.000	ng/mL
101	Chloroxuron	12.583	245.00 > 182.00	61229	14993	100.000	ng/mL
102	Tridiphane	12.599	187.00 > 159.00	31645	10760	100.000	ng/mL
103	Fenchlorphos	12.628	285.00 > 270.00	94510	31095	100.000	ng/mL
104	Demeton_S_methylsulfc	12.763	169.00 > 125.00	32848	7728	100.000	ng/mL
105	Terbutryn	12.949	241.00 > 170.00	42492	14038	100.000	ng/mL
106	Dithiopyr	12.956	354.00 > 286.00	78059	26382	100.000	ng/mL
107	Spiroxamine_1	12.981	100.00 > 72.00	70704	22697	100.000	ng/mL
108	Fenitrothion	13.044	125.00 > 79.00	35350	10741	100.000	ng/mL
109	Bromacil	13.065	207.00 > 190.00	44960	11018	100.000	ng/mL
110	Pirimiphos-methyl	13.119	290.00 > 125.00	40179	13826	100.000	ng/mL
111	Ethofumesate	13.144	161.00 > 105.00	76209	25692	100.000	ng/mL
112	Esprocarb	13.169	222.00 > 91.00	221708	73500	100.000	ng/mL
113	Dimethylvinphos-(E)	13.243	295.00 > 109.00	60031	19169	100.000	ng/mL
114	Dichlofluanid	13.305	123.00 > 77.00	184949	61717	100.000	ng/mL
115	Malathion	13.408	127.00 > 99.00	106643	35240	100.000	ng/mL
116	Aldrin	13.500	263.00 > 193.00	19905	6750	100.000	ng/mL
117	Diethofencarb	13.544	124.00 > 96.00	58265	17897	100.000	ng/mL
118	Metolachlor	13.591	162.00 > 133.00	401865	108726	100.000	ng/mL
119	Aspon	13.626	211.00 > 115.00	128428	43235	100.000	ng/mL
120	Fenthion	13.675	278.00 > 109.00	73797	24961	100.000	ng/mL
121	Dimethylvinphos-(Z)	13.694	295.00 > 109.00	64438	20284	100.000	ng/mL
122	Thiazopyr	13.697	327.00 > 277.00	16105	5694	100.000	ng/mL
123	Cyanazine	13.732	225.00 > 189.00	13404	4732	100.000	ng/mL

ID#	Name	R.Time	m/z	Area	Height	Conc.	Conc.Unit
124	Chlorpyrifos	13.749	197.00 > 169.00	67534	21474	100.000	ng/mL
125	Parathion-ethyl	13.767	291.00 > 109.00	8837	2928	100.000	ng/mL
126	Dicofol	13.807	139.00 > 111.00	321566	76712	100.000	ng/mL
127	Triadimefon	13.847	208.00 > 181.00	41790	11198	100.000	ng/mL
128	Chlorthal-dimethyl	13.905	301.00 > 223.00	75273	25135	100.000	ng/mL
129	Nitrothal-isopropyl	14.023	236.00 > 194.00	69313	23527	100.000	ng/mL
130	Tetraconazole	14.043	336.00 > 156.00	10717	2884	100.000	ng/mL
131	Chlorthion	14.074	125.00 > 79.00	37931	12346	100.000	ng/mL
132	Fenson	14.100	141.00 > 77.00	363031	94500	100.000	ng/mL
133	Flurochloridone	14.125	311.00 > 174.00	29308	9046	100.000	ng/mL
134	Fthalide	14.216	272.00 > 243.00	48280	14508	100.000	ng/mL
135	Pyracabolid	14.236	125.00 > 107.00	36276	8646	100.000	ng/mL
136	MGK-264_1	14.260	164.00 > 93.00	32837	11500	100.000	ng/mL
137	Fosthiazate-1	14.298	195.00 > 103.00	13775	4688	100.000	ng/mL
138	Butralin	14.303	266.00 > 220.00	14319	4706	100.000	ng/mL
139	Bromophos-methyl	14.314	331.00 > 316.00	67732	22228	100.000	ng/mL
140	Diphenamid	14.344	167.00 > 152.00	236151	72516	100.000	ng/mL
141	Fosthiazate-2	14.384	195.00 > 103.00	13281	4450	100.000	ng/mL
142	Pirimiphos-ethyl	14.513	318.00 > 166.00	15833	5350	100.000	ng/mL
143	Cyprodinil	14.568	224.00 > 208.00	104025	30071	100.000	ng/mL
144	Isopropalin	14.568	280.00 > 238.00	65269	23057	100.000	ng/mL
145	Isofenphos-methyl	14.628	199.00 > 121.00	122395	39773	100.000	ng/mL
146	MGK-264_2	14.641	164.00 > 98.00	85228	27429	100.000	ng/mL
147	Metazachlor	14.760	209.00 > 133.00	109155	34201	100.000	ng/mL
148	Dimethametryn	14.810	212.00 > 122.00	83575	26043	100.000	ng/mL
149	Heptachlor-epoxide	14.814	353.00 > 263.00	19066	6551	100.000	ng/mL
150	Pendimethalin	14.819	252.00 > 162.00	25764	9233	100.000	ng/mL
151	Ethychlozate	14.867	165.00 > 102.00	35451	6695	100.000	ng/mL
152	Penconazole	14.851	159.00 > 123.00	78256	19818	100.000	ng/mL
153	Pyrifenox 1	14.966	171.00 > 100.00	15373	4070	100.000	ng/mL
154	Chlozolinate	14.987	187.00 > 124.00	43120	15073	100.000	ng/mL
155	Tolylfluanid	14.998	137.00 > 91.00	162651	51893	100.000	ng/mL
156	Allethrin-1	15.063	123.00 > 81.00	8064	3199	100.000	ng/mL
157	Captan	15.081	149.00 > 70.00	6984	2408	100.000	ng/mL
158	Isofenphos	15.147	213.00 > 121.00	74192	25253	100.000	ng/mL
159	Fluopyram	15.160	173.00 > 145.00	225350	64210	100.000	ng/mL
160	Allethrin-2	15.163	123.00 > 81.00	70125	14592	100.000	ng/mL
161	Fipronil	15.183	367.00 > 213.00	11725	4365	100.000	ng/mL
162	Dimepiperate	15.189	119.00 > 91.00	226520	68877	100.000	ng/mL
163	Mecarbam	15.208	97.00 > 65.00	24300	5284	100.000	ng/mL
164	Quinalphos	15.218	146.00 > 118.00	145192	43810	100.000	ng/mL
165	Triadimenol	15.217	168.00 > 70.00	26369	7184	100.000	ng/mL
166	Phenthoate	15.240	274.00 > 121.00	36087	11626	100.000	ng/mL
167	Folpet	15.303	104.00 > 76.00	60249	17690	100.000	ng/mL
168	Zoxamide	15.353	187.00 > 159.00	6706	1758	100.000	ng/mL
169	Procymidone	15.415	96.00 > 67.00	296643	94224	100.000	ng/mL

ID#	Name	R.Time	m/z	Area	Height	Conc.	Conc.Unit
170	Chlorbenside	15.435	125.00 > 99.00	124682	27809	100.000	ng/mL
171	Crotoxyphos	15.433	193.00 > 127.00	15480	4977	100.000	ng/mL
172	Chinomethionat	15.552	206.00 > 148.00	156226	33118	100.000	ng/mL
173	Chlorflurenol_methyl	15.544	215.00 > 152.00	231119	69843	100.000	ng/mL
174	Triflumizole	15.551	206.00 > 179.00	25433	6635	100.000	ng/mL
175	Chlordane_2	15.611	373.00 > 264.00	50803	15956	100.000	ng/mL
176	Methidathion	15.671	145.00 > 85.00	46799	13820	100.000	ng/mL
177	Bromophos-ethyl	15.775	359.00 > 303.00	60749	20212	100.000	ng/mL
178	Paclobutrazole	15.833	236.00 > 125.00	34228	9324	100.000	ng/mL
179	Pyrifenox 2	15.842	171.00 > 100.00	28470	7148	100.000	ng/mL
180	Fenothiocarb	15.877	161.00 > 72.00	8478	1988	100.000	ng/mL
181	Endosulfan_alphs	16.023	195.00 > 159.00	16625	5249	100.000	ng/mL
182	Tetrachlorvinphos	16.044	329.00 > 109.00	54308	17491	100.000	ng/mL
183	Chlordane_1	16.127	373.00 > 264.00	41117	13346	100.000	ng/mL
184	Butachlor	16.191	176.00 > 147.00	65909	20776	100.000	ng/mL
185	Flumetralin	16.218	143.00 > 107.00	70582	23376	100.000	ng/mL
186	Flutriafol	16.258	123.00 > 95.00	101512	26289	100.000	ng/mL
187	TCMTB	16.299	180.00 > 136.00	74023	10237	100.000	ng/mL
188	Nonachlor_trans	16.294	409.00 > 300.00	40373	12804	100.000	ng/mL
189	Chlorfenson	16.375	175.00 > 111.00	384546	98817	100.000	ng/mL
190	Chlorfluazuron	16.381	321.00 > 304.00	6707	2491	100.000	ng/mL
191	Fenamiphos	16.434	303.00 > 288.00	4524	1741	100.000	ng/mL
192	Napropamide	16.462	128.00 > 72.00	138860	39099	100.000	ng/mL
193	Diethatyl-ethyl	16.470	188.00 > 160.00	137195	44304	100.000	ng/mL
194	Picoxystrobin	16.502	145.00 > 102.00	134203	46780	100.000	ng/mL
195	Hexaconazole	16.513	83.00 > 82.00	88579	21425	100.000	ng/mL
196	Fluorodifen	16.543	190.00 > 146.00	12714	4113	100.000	ng/mL
197	Flutolanil	16.585	173.00 > 145.00	609799	157326	100.000	ng/mL
198	Imazalil	16.647	215.00 > 173.00	15467	3958	100.000	ng/mL
199	Prothiofos	16.667	267.00 > 239.00	50525	17271	100.000	ng/mL
200	Isoprothiolane	16.713	162.00 > 134.00	55648	17600	100.000	ng/mL
201	Profenofos	16.769	339.00 > 269.00	26882	9001	100.000	ng/mL
202	Fludioxonil	16.835	248.00 > 182.00	57840	11590	100.000	ng/mL
203	Uniconazole	16.854	234.00 > 165.00	12627	3582	100.000	ng/mL
204	DDE_pp	16.885	246.00 > 176.00	178245	56751	100.000	ng/mL
205	Pretilachlor	16.893	238.00 > 162.00	12417	3586	100.000	ng/mL
206	Tribufos	16.901	169.00 > 57.00	54396	15924	100.000	ng/mL
207	Dieldrin	16.905	263.00 > 193.00	12367	3928	100.000	ng/mL
208	Oxadiazon	17.084	258.00 > 175.00	70146	23747	100.000	ng/mL
209	Iprovalicarb-1	17.100	134.00 > 42.00	19945	6194	100.000	ng/mL
210	Myclobutanil	17.160	179.00 > 125.00	123426	25904	100.000	ng/mL
211	Diclobutrazole	17.245	272.00 > 161.00	14836	4442	100.000	ng/mL
212	Aramit-1	17.254	175.00 > 135.00	8383	2869	100.000	ng/mL
213	Flusilazole	17.285	233.00 > 165.00	28113	7393	100.000	ng/mL
214	Oxyflofen	17.281	361.00 > 317.00	2346	900	100.000	ng/mL
215	Azaconazole	17.311	217.00 > 173.00	176450	36895	100.000	ng/mL

ID#	Name	R.Time	m/z	Area	Height	Conc.	Conc.Unit
216	Methoprotryne	17.313	256.00 > 212.00	32698	9800	100.000	ng/mL
217	Bupirimate	17.406	273.00 > 193.00	27138	8208	100.000	ng/mL
218	Thifluzamide	17.437	166.00 > 125.00	123906	37533	100.000	ng/mL
219	Iprovalicarb-2	17.450	134.00 > 42.00	23211	7529	100.000	ng/mL
220	Kresoxim-methyl	17.459	116.00 > 89.00	217663	67953	100.000	ng/mL
221	Isoxanthion	17.589	177.00 > 130.00	13283	4355	100.000	ng/mL
222	Aramit-2	17.254	175.00 > 135.00	8383	2869	100.000	ng/mL
223	Cyproconazole-1	17.635	139.00 > 111.00	62715	11800	100.000	ng/mL
224	Cyproconazole-2	17.626	139.00 > 111.00	59619	11564	100.000	ng/mL
225	Endrin	17.959	263.00 > 191.00	3304	1053	100.000	ng/mL
226	Cyflufenamid	17.775	91.00 > 65.00	122478	28065	100.000	ng/mL
227	Perthane	17.775	223.00 > 193.00	74109	23606	100.000	ng/mL
228	Fluazifop_butyl	17.825	282.00 > 238.00	55528	16627	100.000	ng/mL
229	Chlorfenapyr	17.846	137.00 > 102.00	10961	3082	100.000	ng/mL
230	Fenoxanil	17.864	189.00 > 125.00	51542	10395	100.000	ng/mL
231	Methyltrithion	17.896	125.00 > 79.00	17422	5079	100.000	ng/mL
232	Endosulfan_beta	17.963	195.00 > 159.00	12974	4223	100.000	ng/mL
233	Chlorobenzilate	18.021	251.00 > 139.00	206942	58571	100.000	ng/mL
234	Chloropropylate	18.022	139.00 > 111.00	428111	104856	100.000	ng/mL
235	Flufenpyr_ethyl	18.192	408.00 > 345.00	20846	6945	100.000	ng/mL
236	Diniconazole	18.200	268.00 > 232.00	16696	5214	100.000	ng/mL
237	Etaconazole_1	18.225	173.00 > 145.00	33398	7740	100.000	ng/mL
238	DDD_pp	18.320	235.00 > 165.00	323772	99153	100.000	ng/mL
239	Flamprop-isopropyl	18.336	105.00 > 77.00	708372	216120	100.000	ng/mL
240	Etaconazole_2	18.359	173.00 > 145.00	56233	11128	100.000	ng/mL
241	Nonachlor_cis	18.425	409.00 > 300.00	25490	7805	100.000	ng/mL
242	DDT_op	18.430	235.00 > 165.00	265647	81382	100.000	ng/mL
243	Oxadixyl	18.480	163.00 > 132.00	125013	36040	100.000	ng/mL
244	Penthiopyrad	18.526	177.00 > 101.00	57099	15403	100.000	ng/mL
245	Ethion	18.547	231.00 > 129.00	53700	16435	100.000	ng/mL
246	Benodanil	18.617	231.00 > 203.00	199660	31991	100.000	ng/mL
247	Chlorthiophos_1	18.212	325.00 > 269.00	9330	3044	100.000	ng/mL
248	Chlorthiophos_2	18.654	325.00 > 269.00	48385	15872	100.000	ng/mL
249	Tetrasul	18.692	324.00 > 254.00	69659	20438	100.000	ng/mL
250	Mepronil	18.799	119.00 > 91.00	550674	127933	100.000	ng/mL
251	Sulprofos	18.924	156.00 > 141.00	68100	21391	100.000	ng/mL
252	Penflufen	18.929	274.00 > 141.00	107071	30114	100.000	ng/mL
253	Triazophos	18.987	161.00 > 134.00	25240	6947	100.000	ng/mL
254	Chlornitrofen	19.121	317.00 > 287.00	13405	4226	100.000	ng/mL
255	Isoxadifen-ethyl	19.120	222.00 > 204.00	44183	14007	100.000	ng/mL
256	Ofurace	19.144	232.00 > 158.00	74089	22078	100.000	ng/mL
257	Carbophenothion	19.232	121.00 > 65.00	29827	9296	100.000	ng/mL
258	Benalaxyl	19.327	148.00 > 105.00	120136	35189	100.000	ng/mL
259	Edifenphos	19.373	173.00 > 109.00	140019	40605	100.000	ng/mL
260	Quinoxyfen	19.385	237.00 > 208.00	207934	56611	100.000	ng/mL
261	Endosulfan_sulfate	19.449	272.00 > 165.00	1440	466	100.000	ng/mL

ID#	Name	R.Time	m/z	Area	Height	Conc.	Conc.Unit
262	Propiconazole-1	19.494	259.00 > 191.00	3995	1130	100.000	ng/mL
263	Norflurazon	19.520	303.00 > 145.00	61623	11198	100.000	ng/mL
264	DDT_pp	19.605	235.00 > 165.00	232446	71235	100.000	ng/mL
265	Isotianil	19.650	180.00 > 91.00	90663	16826	100.000	ng/mL
266	Propiconazole-2	19.714	259.00 > 191.00	10004	2241	100.000	ng/mL
267	Pyriminobac-methyl(E)	19.938	302.00 > 256.00	58811	17914	100.000	ng/mL
268	Tebuconazole	20.067	125.00 > 89.00	29328	6348	100.000	ng/mL
269	Nuarimol	20.087	139.00 > 111.00	59474	15712	100.000	ng/mL
270	Diclofop_methyl	20.233	340.00 > 253.00	62860	18169	100.000	ng/mL
271	Diflufenican	20.357	394.00 > 266.00	64715	18859	100.000	ng/mL
272	Piperonyl butoxide	20.490	176.00 > 131.00	60800	18941	100.000	ng/mL
273	Epoxiconazole	20.696	192.00 > 138.00	65675	16995	100.000	ng/mL
274	Mefenpyr-diethyl	20.915	253.00 > 189.00	103363	30569	100.000	ng/mL
275	Benzoylprop_ethyl	21.020	105.00 > 77.00	806015	235344	100.000	ng/mL
276	Spiromesifen	21.042	272.00 > 209.00	15924	5146	100.000	ng/mL
277	Iprodione	21.076	187.00 > 124.00	28516	7341	100.000	ng/mL
278	Pyridaphenthion	21.229	340.00 > 199.00	7247	2508	100.000	ng/mL
279	Phosmet(PMP)	21.267	160.00 > 77.00	143672	39130	100.000	ng/mL
280	Tetramethrin-1	21.304	164.00 > 107.00	22250	7258	100.000	ng/mL
281	Bromopropylate	21.385	183.00 > 155.00	92769	24447	100.000	ng/mL
282	EPN	21.412	169.00 > 141.00	80061	23952	100.000	ng/mL
283	Fenoxycarb	21.445	255.00 > 186.00	48915	6456	100.000	ng/mL
284	Picolinafen	21.529	376.00 > 238.00	40278	10462	100.000	ng/mL
285	Tetramethrin-2	21.552	164.00 > 107.00	79577	25065	100.000	ng/mL
286	Bifenthrin	21.578	181.00 > 166.00	388174	120390	100.000	ng/mL
287	Piperophos	21.618	320.00 > 122.00	17541	5636	100.000	ng/mL
288	Methoxychlor	21.649	227.00 > 169.00	95216	28402	100.000	ng/mL
289	Fenpropathrin	21.772	97.00 > 55.00	91802	28346	100.000	ng/mL
290	Etoxazole	21.859	141.00 > 113.00	80761	16032	100.000	ng/mL
291	Tebufenpyrad	21.882	333.00 > 171.00	52820	16043	100.000	ng/mL
292	Fenamidone	21.883	238.00 > 103.00	16501	5873	100.000	ng/mL
293	Metconazole	21.922	125.00 > 89.00	14022	3825	100.000	ng/mL
294	Indanofan	21.917	174.00 > 159.00	87709	23957	100.000	ng/mL
295	Fenazaquin	21.944	145.00 > 117.00	477829	105653	100.000	ng/mL
296	Bifenox	22.017	341.00 > 310.00	7790	2599	100.000	ng/mL
297	Anilofos	22.098	226.00 > 157.00	37152	11870	100.000	ng/mL
298	Clomeporp	22.129	148.00 > 120.00	26019	6685	100.000	ng/mL
299	Tetradifon	22.292	111.00 > 75.00	111571	30188	100.000	ng/mL
300	Phenothrin	22.483	123.00 > 81.00	48423	15138	100.000	ng/mL
301	Furathiocarb	22.531	163.00 > 107.00	99326	31267	100.000	ng/mL
302	Flutamone	22.548	333.00 > 120.00	29403	6648	100.000	ng/mL
303	Azinphos-methyl	22.631	160.00 > 132.00	50045	13549	100.000	ng/mL
304	Phosalone	22.648	182.00 > 111.00	64667	19734	100.000	ng/mL
305	Pentoxazon	22.681	285.00 > 70.00	104958	32766	100.000	ng/mL
306	Leptophos	22.722	377.00 > 362.00	35973	11262	100.000	ng/mL
307	Mirex	22.906	272.00 > 237.00	208247	59679	100.000	ng/mL

ID#	Name	R.Time	m/z	Area	Height	Conc.	Conc.Unit
308	Mefenacet	23.021	192.00 > 136.00	147194	26085	100.000	ng/mL
309	Cyhalofop-butyl	23.070	256.00 > 120.00	119713	25678	100.000	ng/mL
310	Cyhalothrin-1	23.133	208.00 > 181.00	33693	10830	100.000	ng/mL
311	Acrinathrin_1	23.508	208.00 > 181.00	10416	3230	100.000	ng/mL
312	Cyhalothrin-2	23.507	208.00 > 181.00	15925	5159	100.000	ng/mL
313	Fenarimol	23.572	139.00 > 111.00	85457	20843	100.000	ng/mL
314	Lactofen	23.620	223.00 > 132.00	3690	1206	100.000	ng/mL
315	Azinphos-ethyl	23.867	160.00 > 132.00	55923	15339	100.000	ng/mL
316	Pyrazophos	23.896	221.00 > 193.00	32177	9588	100.000	ng/mL
317	Acrinathrin_2	23.930	208.00 > 181.00	23828	7405	100.000	ng/mL
318	Pyraclofos	24.106	194.00 > 138.00	28078	6906	100.000	ng/mL
319	Dialifor	24.102	208.00 > 89.00	13661	4175	100.000	ng/mL
320	Metrafenone	24.211	393.00 > 362.00	11283	3170	100.000	ng/mL
321	Permethrin-1	24.824	183.00 > 153.00	28245	8658	100.000	ng/mL
322	Pyridaben	25.019	147.00 > 117.00	251220	72575	100.000	ng/mL
323	Permethrin-2	25.075	183.00 > 153.00	41797	12109	100.000	ng/mL
324	Coumaphos	25.202	362.00 > 109.00	7683	2296	100.000	ng/mL
325	Prochloraz	25.320	180.00 > 138.00	5202	1346	100.000	ng/mL
326	Butafenacil	25.682	331.00 > 180.00	118483	33952	100.000	ng/mL
327	Fenbuconazole	25.884	198.00 > 129.00	89205	15722	100.000	ng/mL
328	Cyfluthrin-1	25.997	163.00 > 127.00	9195	3011	100.000	ng/mL
329	Cyfluthrin-2	26.180	163.00 > 127.00	13187	3931	100.000	ng/mL
330	Cyfluthrin-3	26.327	163.00 > 127.00	9255	2677	100.000	ng/mL
331	Cyfluthrin-4	26.404	163.00 > 127.00	11353	3274	100.000	ng/mL
332	Cypermethrin-1	26.584	163.00 > 127.00	11622	3452	100.000	ng/mL
333	Halfenprox	26.657	263.00 > 115.00	27348	7870	100.000	ng/mL
334	Cypermethrin-2	26.777	163.00 > 127.00	18691	5607	100.000	ng/mL
335	Cypermethrin-3	26.915	163.00 > 127.00	7363	2074	100.000	ng/mL
336	Cypermethrin-4	26.992	163.00 > 127.00	9067	2739	100.000	ng/mL
337	Flucythrinate_1	27.019	199.00 > 157.00	34829	10156	100.000	ng/mL
338	Etofenprox	27.128	163.00 > 135.00	389060	102282	100.000	ng/mL
339	Pyridalyl	27.258	204.00 > 176.00	38657	9279	100.000	ng/mL
340	Flucythrinate_2	27.399	199.00 > 157.00	30913	8994	100.000	ng/mL
341	Silafluofen	27.440	286.00 > 258.00	109348	32379	100.000	ng/mL
342	Flutianil	27.768	231.00 > 216.00	10746	2497	100.000	ng/mL
343	Pyrimidifen	27.988	184.00 > 169.00	59953	12186	100.000	ng/mL
344	Fenvalerate-1	28.317	167.00 > 125.00	61155	17286	100.000	ng/mL
345	Flumioxazine	28.351	354.00 > 326.00	2395	712	100.000	ng/mL
346	Fenvalerate-2	28.713	167.00 > 125.00	16148	4454	100.000	ng/mL
347	Fluvalinate-1	28.734	250.00 > 55.00	19534	5684	100.000	ng/mL
348	Fluvalinate-2	28.859	250.00 > 55.00	15129	4505	100.000	ng/mL
349	Difenoconazole-1	29.063	323.00 > 265.00	12275	2896	100.000	ng/mL
350	Difenoconazole-2	29.191	323.00 > 265.00	17861	3381	100.000	ng/mL
351	Tralomethrin-1	29.328	181.00 > 152.00	279	75	100.000	ng/mL
352	Deltamethrin-1	29.304	181.00 > 152.00	587	127	100.000	ng/mL
353	Indoxacarb	29.707	203.00 > 134.00	58168	13434	100.000	ng/mL

ID#	Name	R.Time	m/z	Area	Height	Conc.	Conc.Unit
354	Tralomethrin-2	29.724	181.00 > 152.00	21815	5258	100.000	ng/mL
355	Deltamethrin-2	29.724	181.00 > 152.00	24588	6331	100.000	ng/mL
356	Flumiclorac_pentyl	30.035	318.00 > 260.00	5093	1395	100.000	ng/mL
357	Tolfenpyrad	30.430	383.00 > 171.00	6103	1292	100.000	ng/mL
358	Cinidone_ethyl	31.615	358.00 > 330.00	44325	8312	100.000	ng/mL
359	Fluthiacet_methyl	31.881	405.00 > 56.00	1449	272	100.000	ng/mL

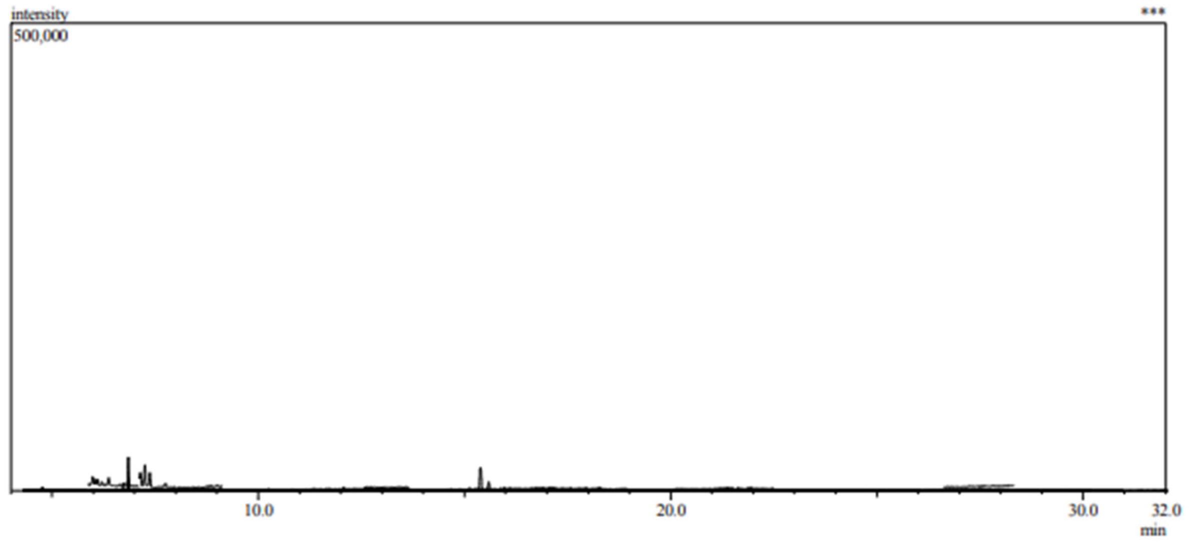

(A)

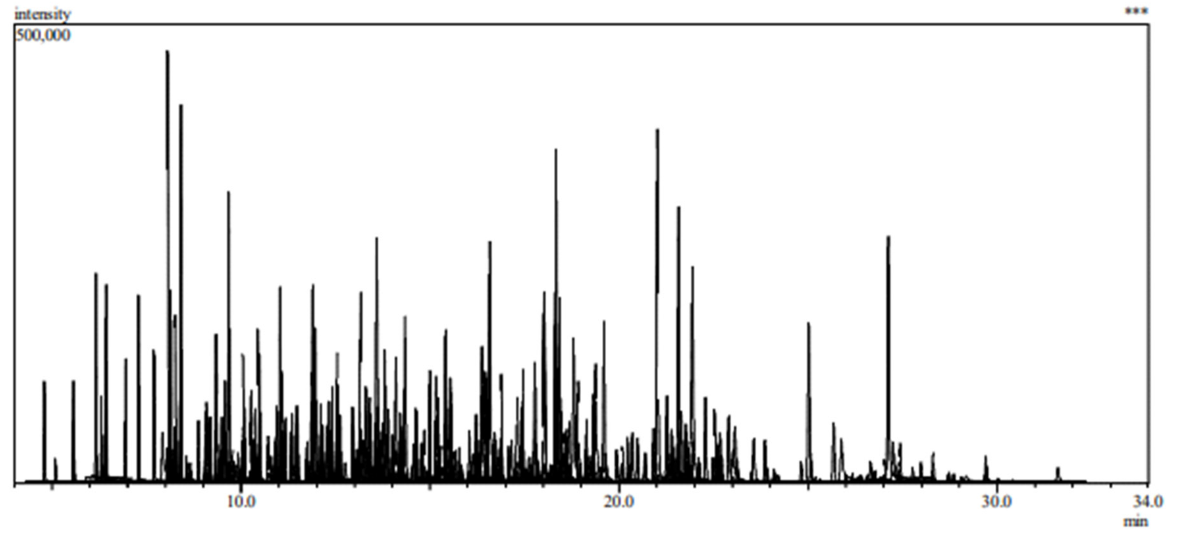

(B)

Figure S3. Representative chromatogram of Chlorpyrifos in corresponding to: (A) control sample, (B) 0.01 mg/L matrix matched standard.

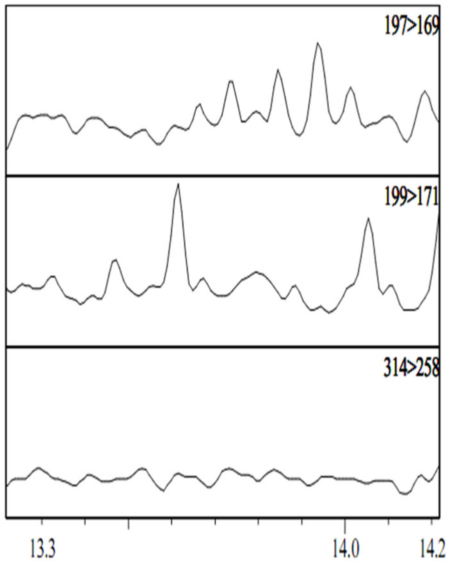

(A)

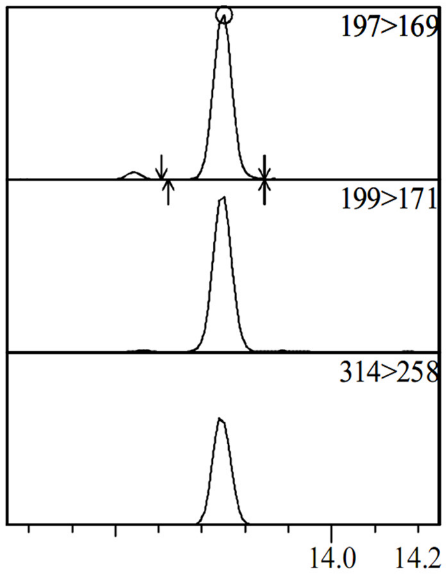

(B)

Figure S4. Representative chromatogram of Chlorpyrifos in corresponding to : (A) control sample, (B) 0.01 mg/L matrix matched standard.

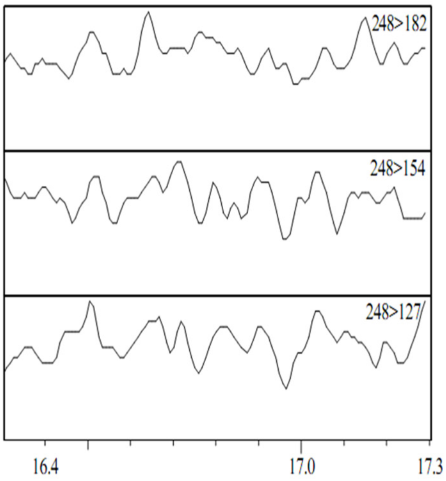

(A)

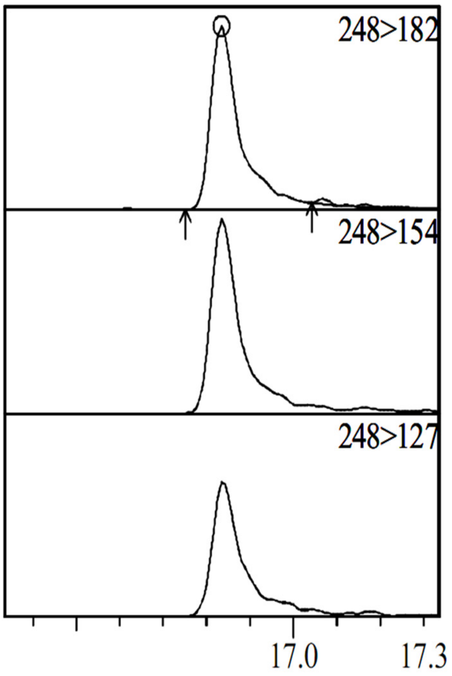

(B)

Figure S5. Representative chromatogram of Fludioxonil in corresponding to : (A) control sample, (B) 0.01 mg/L matrix matched standard.

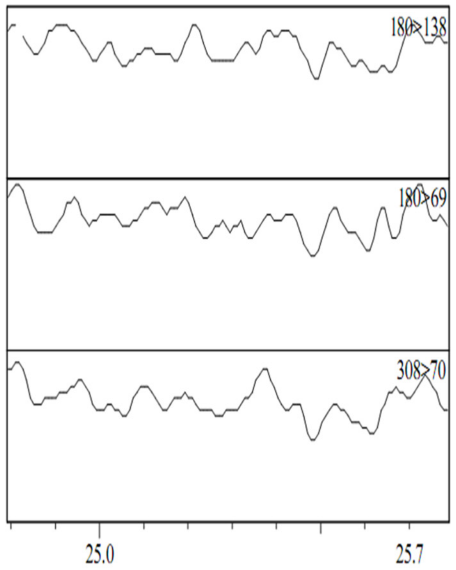

(A)

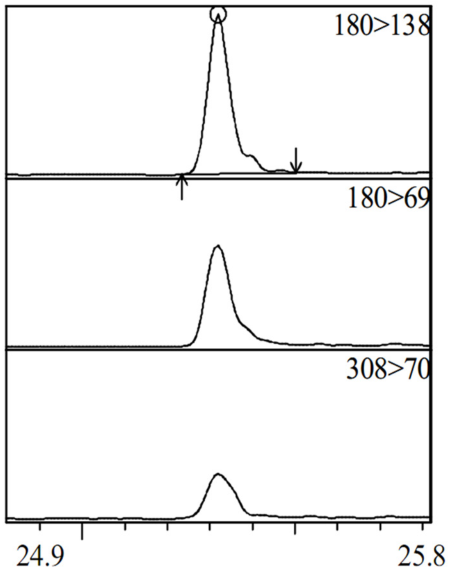

(B)

Figure S6. Representative chromatogram of Prochloraz in corresponding to : (A) control sample, (B) 0.01 mg/L matrix matched standard.
